# Sodium chloride enhances suberization in seminal roots but does not affect cutinized leaf barriers in cultivated and wild barley

**DOI:** 10.1007/s00425-025-04743-9

**Published:** 2025-06-15

**Authors:** Paul Grünhofer, Priya Dharshini Thangamani, Lukas Schreiber, Tino Kreszies

**Affiliations:** 1 Department of Ecophysiology, Institute of Cellular and Molecular Botany, University of Bonn, Kirschallee 1, 53115 Bonn, Germany; 2 Department of Plant Cell Biology, Botanical Institute and Botanical Garden, University of Kiel, Am Botanischen Garten 5, 24118 Kiel, Germany; 3 Centre for Crop Systems Analysis, Wageningen University and Research, Wageningen, The Netherlands

**Keywords:** Abiotic stress, Apoplastic barrier, Cuticle, Exodermis, *Hordeum*, Leaf cuticular wax, Root endodermal suberin, Salinity

## Abstract

**Main conclusion:**

In the two compared barley genotypes, broader genetic variation did not result in a higher salt tolerance. Instead, specific traits like an exodermis might represent valuable future breeding targets.

**Abstract:**

Soil salinification is a globally increasing phenomenon threatening agricultural yields. In this study, we investigated the physiological reactions of two genotypes of the fourth most abundant cereal crop barley in response to hydroponic sodium chloride exposure. It was of interest to compare a modern cultivar intentionally bred for the highest yields with a wild accession comprising a wider genetic background. Since barley is known to be a relatively salt-tolerant crop, three different sodium concentrations of up to 280 mM have been tested. The physiological adaptations of shoots and roots were investigated utilizing stomatal conductance measurements, chlorophyll fluorometry, morphometry, osmotic potential determination, mineral element concentration measurement, as well as histochemical and chemical analysis of apoplastic leaf and root barriers. While the leaf cuticle of both genotypes hardly reacted to the imposed stresses, the roots exhibited an increased endodermal suberization of especially the root tip, which strongly deviated from the previous findings about pure osmotic stress exposure. Interestingly, the putatively higher drought-tolerant wild accession did not show a considerably better growth performance, which in the context of sodium chloride stress might be attributed to its overall significantly smaller endodermal suberization reaction. We conclude that a subsequent study of a wild accession and/or a modern cultivar known to develop an exodermis might deliver valuable additional insights into potential future breeding targets. Such a suberized exodermis might be capable of conveying increased tolerance to toxic salts without negatively affecting water uptake.

**Supplementary Information:**

The online version contains supplementary material available at 10.1007/s00425-025-04743-9.

## Introduction

Some of the major means of a plant to protect its internal tissues from the surrounding environment are the apoplastic barriers of shoots and roots. While the cutinized and wax-impregnated cuticle resides on all primary aerial organs (Kunst and Samuels 2003), the primary subterranean roots are characterized by a suberized endodermis and sometimes also exhibit a nonobligatory suberized exodermis (Eshel and Beeckman 2013). Both types of barriers fulfill different functions, because the highly water-deficient atmosphere establishes the need for a strong restriction of passive water loss from shoots, while the usually water-bearing rhizosphere exposes the roots to all kinds of nutrients and contaminants demanding precise control of uptake or exclusion, respectively (Grünhofer and Schreiber 2023). Thus, it has previously been discovered that it is especially the cuticular waxes (rather than cutin) that convey the cuticle’s water loss resistance (Schönherr 1976) and hypothesized that endo- and exodermal suberization (especially by the aliphatic rather than the aromatic suberin fraction) might help to decrease the plasma membrane surface area available for ion absorption (Enstone et al. 2003; Grünhofer et al. 2024). The latter might be of utmost importance once the plant roots are confronted with high concentrations of salt ions, for example, as a consequence of global climate change.

Naturally occurring salinity or salt stress is the result of a complex interaction of different salt cations, such as calcium (Ca^2+^), magnesium (Mg^2+^), sodium (Na^+^), but also their anions chloride (Cl^−^), sulfate (SO_4_^2−^), and carbonate (CO_3_^2−^) in the root medium (Szabolcs 1989; Zörb et al. 2019). Salinity is defined based on the electrical conductivity (EC) of the saturated paste extract (ECe), which is equivalent to the concentration of salts in a hydroponic solution (Munns and Tester 2008), higher than 4 dS m^−1^, and, on a larger scale, it is currently affecting 10.7% of the total global land area (FAO 2024) where it negatively affects both tree and food crop yields each year (Challinor et al. 2014; Polle and Chen 2015). What sets salinity apart from pure osmotic stress, which is usually defined using the osmotic potential described in megapascal (MPa), is a slower secondary ionic phase leading to ion toxicity in addition to the also occurring rapid initial osmotic phase, which severely affects the water homeostasis (Munns 2002). Out of the above-mentioned salt types, sodium chloride (NaCl) represents the most abundant as it is readily released by weathering from parental rocks and deposited by rainwater carrying oceanic salts (Munns and Tester 2008), which is why ‘salinity’ or ‘salt stress’ will be used as a synonym for exclusive NaCl exposure in this study. In an ideal hydroponic solution, the addition of 20 mmol l^−1^ (mM) of NaCl would lead to a total concentration of 40 mOsmol l^−1^ solutes (based on a complete dissociation of NaCl into Na^+^ and Cl^−^), resulting in an osmotic potential of −0.1 MPa and an ECe of 2 dS m^−1^ (Munns et al. 2020). Thus, the concentration of NaCl in hydroponics and the ECe are directly correlated (10 mM = 1 dS m^−1^), and five categories of no (< 20 mM), low (20–40 mM), medium (40–80 mM), high (80–160 mM), and extreme (> 160 mM) salt stress intensity can be defined for most plants (Polle and Chen 2015).

Interestingly, different species of plants can cope with different NaCl intensities very well. Wild sea barley (*Hordeum marinum*), for example, can withstand concentrations of at least 450 mM (equal to the salinity of seawater) by efficiently excluding both of its dissociated ions (Na^+^ and Cl^−^) as long as possible (Garthwaite et al. 2005). Even commonly cultivated barley (*Hordeum* *vulgare*) from the same genus is known to be relatively salt-tolerant in comparison to Arabidopsis, rice, or wheat (Munns and Tester 2008). Nonetheless, many of our modern agricultural crop plants, including barley, have continuously been bred for the highest possible yields while being cultivated on arable land, which will most certainly have exerted an evolutionary pressure leading to increased salt sensitivity over time (Zörb et al. 2019). With global salinification on the rise, it might be increasingly advisable to consider either the direct cultivation of more salt-tolerant (halophytic) genotypes, potentially exhibiting lower yields (Panta et al. 2014), or the (re-) introduction of still wilder landraces into breeding programs of our modern salt-sensitive (glycophytic) crops (Ismail and Horie 2017).

Globally, barley represents the fourth most abundant cereal crop after wheat, maize, and rice (Mayer et al. 2012). This, at least in part, is owing to its necessity for malt production for the beer and whiskey industry, which also are subject to the severe climatic changes of recent times (Xie et al. 2018). The multitude of modern cultivars of *Hordeum* *vulgare* ssp. *vulgare* is derived from its wild progenitor, *Hordeum* *vulgare* ssp. *spontaneum*, which originates from the so-called fertile crescent (Harlan and Zohary 1966). Even after around 10.000 years of domestication (Haas et al. 2019), cultivated and wild varieties can still be crossed and progenies are fully fertile, allowing the transfer of beneficial traits between them (Gunasekera et al. 1994; Ellis et al. 2000). Although belonging to another species, the above-mentioned sea barley (*H.* *marinum*) could serve as an example of such a trait in the context of salinity, because its high salt tolerance (Garthwaite et al. 2005) might in parts be linked to the formation of a suberized exodermis (Kotula et al. 2017), which has normally not been described for barley (Grünhofer et al. 2021b) with rare exceptions (Liu and Kreszies 2023; Reissinger et al. 2003). While many modern barley cultivars have been screened in the context of differential apoplastic barrier formation of shoots and roots in response to undisturbed development (Richardson et al. 2005; Ranathunge et al. 2017), gene mutation (Hen-Avivi et al. 2016; Müller et al. 2023), illumination conditions (Herzig et al. 2025), nutrient deficiencies (Coffey et al. 2018; Armand et al. 2019; Melino et al. 2021), osmotic stress (Kreszies et al. 2019, 2020b), and also NaCl exposure (Fricke et al. 2006; Even et al. 2018; Knipfer et al. 2021; Lu and Fricke 2023), the detailed comparison of cultivated and wild genotypes in regard to physiological abiotic stress adaptations has yet been performed to a lesser extent.

This was done primarily by three previous studies originating from the same laboratory and utilizing the identical experimental pipeline with slight modifications to the focused plant organ or cultivation condition, comparatively investigating the modern cultivar Scarlett and the wild barley accession ICB181243 (termed Pakistan) in reaction to osmotic stress in hydroponics or water withdrawal in soil (Kreszies et al. 2020a; Shellakkutti et al. 2022; Suresh et al. 2024). In summary (Table 1), these studies provided first insights into overall very similar, yet in detail nonetheless remarkably different abiotic stress adaptations of Pakistan, which comprises a wider genetic diversity than its cultivated counterpart Scarlett. In all parameters investigated, Scarlett and Pakistan hardly differed in the direction of their reactions, but more often in their intensities. What more strikingly set Pakistan apart from Scarlett were its consistently longer roots which should be beneficial in accessing deeper and less dehydrated soils during water limitations (Suresh et al. 2024) and its expected higher drought tolerance due to the reduced response of endodermal barriers to osmotic stress indicating further coping mechanisms potentially missing in Scarlett (Kreszies et al. 2020a). Although this hypothesis could not yet be confirmed by additionally evaluating its cuticular barrier properties (Shellakkutti et al. 2022), it was of high interest to further expand the current knowledge with data on hydroponically applied NaCl exposure. Salinity is not only a globally prevalent abiotic stress that is becoming increasingly threatening to agriculture and agroforestry (FAO 2024) but is also known to be even more challenging for the plant than just water limitation alone (Munns and Tester 2008). In roots of the comparably stress-susceptible gray poplar (*Populus* × *canescens*), NaCl exposure was already shown to unfold relatively higher inducibility of a protective exodermis than pure osmotic stress (Grünhofer et al. 2022b), and the present study intended to investigate this circumstance in cultivated and wild barley genotypes.

**Table 1:** Summary table of data generated in studies using the same barley genotypes, performed in the same laboratory, and employing the same standardized experimental pipeline but with different focused plant organs or modified cultivation conditions

	Reference	Kreszies et al. 2019	Kreszies et al. 2020a		Shellakkutti et al. 2022		Suresh et al. 2024		This study	
Study overview	Focused organ	Roots	Roots		Leaves		Roots		Leaves & roots	
	Cultivation condition	Hydroponics	Hydroponics		Hydroponics		Soil		Hydroponics	
	Stress type	Osmotic vs. Control	Osmotic vs. Control		Osmotic vs. Control		Water withdrawal vs. Control		NaCl vs. Control	
	Stress intensity > < = focused on in table	− 0.4 MPa > − 0.8 MPa < − 1.2 MPa	− 0.8 MPa		− 0.8 MPa		−0.4 MPa > −1.0 MPa <		80 mM > 180 mM < 280 mM	
	Cultivar/accession	Scarlett	Scarlett (or Morex)	Pakistan (ICB181243)	Scarlett	Pakistan (ICB181243)	Scarlett	Pakistan (ICB181243)	Scarlett	Pakistan (ICB181243)
Physiology	Shoot or leaf length (%)	–	↘ (17%) S	↘ (26%) S	―	―	→ L1 ↘ (63%) L2 ↘ (100%) L3	↘ (70%) L1 ↘ (34%) L2 ↘ (100%) L3	↘ (43%) S	↘ (39%) S
	Leaf area (%)	―	―	―	↘ (32%) L1 ↘ (60%) L2	↘ (32%) L1 ↘ (61%) L2	―	―	→ L1	→ L1
	Leaf epidermal cell length	―	―	―	↘	↘	―	―	―	―
	Leaf element content	―	―	―	―	―	―	―	(for 80 mM) ↗ Na, P, Fe ↘ Ca, K	(for 80 mM) ↗ Na, P ↘ Ca, K
	Root:shoot dry-weight ratio	―	↗	→	―	―	↗	↗	―	―
	Root length (%)	↘ (16%)	↘ (24%)	↘ (19%)	―	―	↘ (54%)	↘ (52%)	↘ (31%)	↘ (53%)
	Root proline content	―	↗	↗	―	―	―	―	―	―
	Root osmotic potential	―	↘	↘	―	―	―	―	↘	↘
	Root element content	―	―	―	―	―	―	―	(for 80 mM) ↗ Na, Fe ↘Ca, K	(for 80 mM) ↗ Na, Fe ↘Ca, K
Microscopy	Leaf epicuticular wax	―	―	―	↗	↗	―	―	―	―
	Root endodermal Casparian bands	→	→ (not shown)	→ (not shown)	―	―	↗	↗	→ (not shown)	→ (not shown)
	Root lignificatin	―	―	―	―	―	↗ ZB, ZC	↗ ZB, ZC	―	―
	Root suberization	↗ ZB	―	―	―	―	↗ ZA	↗ ZA	↗ ZA, ZB	↗ ZA, ZB
Chemistry	Leaf wax (%)	―	―	―	↗ (39%) SaL1 → SaL2 → L1 ↘ (58%) L2	↗ (42%) SaL1 ↗ (32%) SaL2 → L1 ↘ (51%) L2	―	―	→ SaL1	→ SaL1
	Leaf cutin	―	―	―	→ SaL1 ↗ (40%) SaL2 → L1 → L2	→ SaL1 ↗ (79%) SaL2 ↘ (35%) L1 → L2	―	―	→ SaL1	→ SaL1
	Root lignin	―	―	―	―	―	→ ZA ↗ ZB ↗ ZC	↗ ZA ↗ ZB ↗ ZC	―	―
	Root aliphatic suberin (%)	→ ZA ↗ (117%) ZB ↗ (55%) ZC	→ ZA ↗ (140%) ZB ↗ (39%) ZC	→ ZA → ZB ↗ (45%) ZC	―	―	↗ (144%) ZA ↗ (150%) ZB ↗ (147%) ZC	↗ (75%) ZA ↗ (59%) ZB ↗ (103%) ZC	↗ (426%) ZA ↗ (311%) ZB ↗ (69%) ZC	↗ (119%) ZA ↗ (183%) ZB ↗ (75%) ZC
	Root aromatic suberin	→ ZA → ZB → ZC	→ ZA → ZB → ZC	→ ZA → ZB ↗ ZC	―	―	→ ZA → ZB → ZC	→ ZA → ZB → ZC	↗ ZA ↗ ZB ↗ ZC	→ ZA → ZB ↗ ZC
DEGs	Leaf or root gene expression changes	↗ Suberin → Aquaporin	↗ Suberin ↗ Proline → Aquaporin	→ Suberin ↗ Proline → Aquaporin	↗ Cutin ↗ Wax	↗ Wax	↗ Suberin ↗ Lignin → Aquaporin	↗ Suberin ↗ Lignin → Aquaporin	―	―
Transport	Leaf stomatal conductance	―	―	―	↘	↘	―	―	↘	↘
	Leaf residual transpiration	―	―	―	→	→	―	―	―	―
	Leaf photosynthetic performance	―	―	―	(to 190 PAR) →	(to 190 PAR) →	―	―	(to 820 PAR) →	(to 820 PAR) ↘
	Root hydrostatic hydraulic conductivity	↘	↘	→	―	―	―	―	―	―
	Root osmotic hydraulic conductivity	→	→	→	―	―	―	―	―	―

The barley cultivar Scarlett (*Hordeum* *vulgare* spp. *vulgare*) and the wild accession ICB181243 from Pakistan (spp. *spontaneum*) were compared. ― not investigated, ↗ significant increase, → no change, ↘ significant decrease, DEGs refers to differentially expressed genes; S, L1, L2, and L3 refer to the shoot, the first, second, and third leaves, respectively; SaL1 and SaL2 refers to the relation of wax compounds to the leaf surface area (µg cm^−2^) instead of the whole leaf L1 and L2 (µg); the zones refer to functional endodermal seminal root suberization (ZA = no suberization, 0–25%; ZB = patchy suberization, 25–50%; ZC = full suberization, 50–100% of relative root length); (%) indicates that only for the significant changes (↗ or ↘) of some selected experiments (organ length or area, leaf wax, leaf cutin, and root aliphatic suberin), the calculated relative change (% of stress versus control) is given in parentheses

## Materials and methods

The standardized experimental pipeline of this study comprising plant cultivation, gathering of physiological data, as well as histochemical and analytical analyses of apoplastic transport barriers was carried out as previously already done with barley plants (Kreszies et al. 2019; Shellakkutti et al. 2022) and more thoroughly summarized and methodologically explained on the example of poplar (Grünhofer et al. 2021a).

### Plant material

In all experiments, plants of the barley cultivar Scarlett (*Hordeum* *vulgare* spp. *vulgare*) and the wild accession ICB181243 from Pakistan (*H. vulgare* spp. *spontaneum*) were compared with each other. These two genotypes were selected based on prior detailed characterization comprising (i) an initial screening of the apoplastic root barriers of three wild and three cultivated barley genotypes in response to osmotic stress (Kreszies et al. 2020a), and (ii) two subsequent studies thoroughly examining the apoplastic leaf barriers of both Scarlett and Pakistan in response to osmotic stress (Shellakkutti et al. 2022) and investigating the apoplastic root barriers of both Scarlett and Pakistan in response to water withdrawal in soil (Suresh et al. 2024) (summarized in Table 1). In addition, preliminary experiments had already indicated that an inducible exodermis can form in certain wild cultivars in response to osmotic stress (Kreszies et al. 2020a). Thus, it was of great interest to investigate whether or not this might also be the case for Pakistan when subjected to the even more intense stress conditions of NaCl exposure. All plants were grown from seeds, which had been stratified for at least 1 week at 4 ℃ before being germinated on wet filter paper at 25 ℃ in the dark (day 0).

### Cultivation conditions

After 3 days of germination (day 3), the seedlings were transferred into hydroponic systems filled with aerated half-strength Hoagland nutrient solution (Hoagland and Arnon 1950) located in a climate chamber. The environmental long-day (16 h illumination and 8 h darkness) conditions comprised a light intensity of 130 μmol m^−2^ s^−1^, a temperature of 20–23 ℃, and a relative humidity of 50–65%. Altogether, hydroponic cultivation was carried out until day 12, on which the plants exhibited up to two leaves and five-to-six seminal roots. Since the second leaf was not always adequately developed after the NaCl- stress treatments, all subsequent experiments were performed only with the first leaf. This approach is justifiable, because the previous investigations have shown that during the given experimental conditions, both the first and second leaves yielded highly comparable findings (Shellakkutti et al. 2022).

### Stress treatments

On day 6, after 3 days of germination and 3 days of non-stressed hydroponic cultivation, the plants were divided into up to four subsets for the remaining 6 days. This included continued non-stressed cultivation (Control) and three progressively increasing intensities of NaCl exposure. The aim of these three salinity treatments was to precisely match the previously investigated osmotic potentials of − 0.4, − 0.8, and − 1.2 MPa induced by PEG8000 (Kreszies et al. 2019). The osmotic potentials (Ψ_s_) were matched using the Van’t Hoff equation (*Ψ*_*s*_ = − *R T i C*), in which *R* is the universal gas constant (*R* = 8.314 J mol^−1^ K^−1^), *T* is the absolute temperature in Kelvin (*T* = 298.15 K), *i* is the dissociation factor of NaCl (*i* = 2), and *C* is the osmolarity of the medium. By strictly following this equation, osmolarities of 80, 160, and 240 mM for NaCl were calculated. However, these theoretically estimated concentrations were practically checked using a WP4C Dewpoint PotentiaMeter (Decagon Devices) and subsequently corrected to slightly higher values with increasing concentrations based on the factual dissociation of NaCl, which is rarely 100%, resulting in the employed osmolarities of 80, 180, and 280 mM, respectively.

Not all followingly explained measurements were conducted for both, the shoots and roots of both Scarlett and Pakistan, after treatment with all stress intensities. Instead, the greatest focus was laid on the roots of Scarlett, which was supplemented with data on the shoots of Scarlett, but also with data on the roots and shoots of Pakistan.

### Physiological parameters

Quickly occurring changes in stomatal transpiration and photosynthetic yield were monitored within the first 48 h after stress application (days 6–8). In contrast, more slowly developing effects on shoot and root lengths, leaf areas, root osmotic potentials, and leaf and root macro- and micronutrient profiles were investigated after plant harvest (day 12).

Stomatal transpiration was measured 0.5 (day 6), 24 (day 7), and 48 h (day 8) after NaCl stress application with an AP4 Porometer (Delta-T Devices). The photosynthetic yield (defined as the photochemical quantum yield of photosystem II and calculated based on fluorescence measurements) was tested 48 h (day 8) after NaCl stress application with light curves performed by the Junior-PAM (Walz). The light intensities, given as photosynthetically active radiation (PAR), were increased in eight steps (each step lasted 5 min) between 0 and 820 µmol m^−2^ s^−1^.

While the shoot and root lengths were measured with a ruler after harvesting (day 12), the leaf areas were determined using a flat-bed scanner (Canon Inc.). The determination of root osmotic potentials was done by grinding seminal roots in a Retsch MM400 mixer mill (Retsch GmbH) at a frequency of 30 rounds s^−1^ for 1 min. After centrifugation at 12,300 g for 2 min, the osmolarity of the supernatant was measured with the OSMOMAT 030 freezing point osmometer (gonotec) and inserted into the Van’t Hoff equation (given above) to calculate the corresponding osmotic potential.

### Mineral nutrient analysis

Harvested leaf and root samples were dried in the oven at 60 °C until their weight remained constant before being finely powdered. Using a high-accuracy balance, 100 mg of dried and powdered material was transferred to a Teflon digestion tube. The digestion medium consisted of 4 ml concentrated HNO_3_ and 2 ml 30% H_2_O_2_. The microwave (MLS Mikrowellen-Labor-Systeme GmbH) digestion was performed at 200 °C and 15 bar for 75 min. After digestion, the samples were diluted in 25 ml double-distilled H_2_O. In each batch of microwave digestion, a certified reference material (apple leaf, SRM 1515, National Institute of Standards and Technology) was also digested to ensure high accuracy of the measurements. Nutrient concentrations were measured by Inductively Coupled Plasma Optical Emission Spectrometry (Vista-RL Simultaneous ICP-OES, Varian Inc.) equipped with a Quartz Torch Low Flow with a 1.4 mm injector and a Sea Spray nebulizer with a sample uptake of 2 ml min^−1^. Calibration was achieved by a multielement standard solution (Bernd Kraft).

### Histochemistry

The endodermal deposition of Casparian bands was visualized with 0.1% (w/v) berberine hemi-sulfate as well as 0.5% (w/v) aniline blue (Brundrett et al. 1988), and that of suberin lamellae with 0.01% (w/v) fluorol yellow 088 (Brundrett et al. 1991). The harvested seminal roots were cross-sectioned (30 µm thickness) with a Leica CM1950 cryostat microtome (Leica Biosystems) at representative relative positions of previously established functional developmental zones (Kreszies et al. 2019). This zonation is based on 0% relative root length representing the root tip and 100% representing the root base. When the cultivar Scarlett is grown in non-stress hydroponic conditions, zone A (0–25%) is characterized by no visible suberin deposition, zone B (25–50%) exhibits patchy suberin lamellae formation, and in Zone C (50–100%), the endodermis is fully suberized. After staining of the cross-sections, they were investigated using a UV pE-300lite light source (CoolLED) and a UV filter set (excitation filter BP 365, dichroic mirror FT 395, barrier filter LP 397; Zeiss), and photographed using a Canon EOS 600D camera (Canon Inc.). Picture editing (e.g., cropping, scale bars, and brightness adjustments) has been performed with ImageJ (Abramoff et al. 2004), and thus, color intensity does not reflect suberin quantity. For precise suberin quantification, chemical analyses were carried out.

### Chemical analysis

The quantification of apoplastic leaf (cuticle) and root (endodermis) transport barriers was carried out precisely following the previously stated protocols (Kreszies et al. 2019; Shellakkutti et al. 2022) to enable reliable comparability of data. For more details, the reader is advised to read these publications and refer to Baales et al. (2021).

Very briefly, to extract soluble cuticular waxes, the first leaf of each plant yielded one biological sample and was dipped in 2 ml of chloroform for 10 s before being scanned for later surface area determination. The wax extracts were spiked with 10 μg of tetracosane as an internal standard, and their volume was reduced under a gentle stream of nitrogen. In parallel, the total delipidation of the leaves was achieved by storing them in a frequently renewed 1:1 (v/v) chloroform:methanol solution for 3 weeks. Before the cutin analysis could be executed, the leaves had to be dried on PTFE plates. In contrast to the leaves, the harvested seminal roots were pooled into groups of 10 to form one biological replicate, divided into the three functional developmental zones (0–25, 25–50, 50–100% relative root length) before being enzymatically digested for 3 weeks with 0.5% (w/v) cellulase and 0.5% (w/v) pectinase. After enzymatic digestion, isolated cell walls were washed in borate buffer and then transferred to a frequently renewed 1:1 (v/v) chloroform:methanol solution for soluble lipid extraction for a further 2 weeks. Once this was finished, the roots were also dried on PTFE plates. Both cutin and suberin samples could now be spiked with selected internal standards (cutin: 10 µg tetracosane; suberin: 10 µg dotriacontane) and subsequently be transesterified with BF_3_-methanol to release cutin and suberin monomers. From here on, the cuticular wax, cutin, and suberin samples were derivatized using 20 μl BSTFA (N,O-bis-trimethylsilyltrifluoracetamide) and 20 μl pyridine before being analyzed by gas chromatography and mass spectrometry, incorporating slightly different injection mechanisms and temperature programs for each sample type.

Finally, the estimated cuticular wax and cutin amounts were related to the surface area of the scanned leaves, and the suberin amounts were related to the endodermal surface area (A_EN_) calculated for each root zone by following the equation *A*_*EN*_ = *2π r L* with *r* being the endodermis radius and *L* being the length of the individual root zone.

### Statistical analysis

At least three independent biological replicates were used for each experiment. The data were statistically analyzed with Origin Pro 2021 (OriginLab Corporation). Significant differences between sample groups were evaluated with a one-way ANOVA with Fisher’s LSD post hoc test (*P* < 0.05) and indicated by differential letters. The data are visualized by means and standard deviations.

## Results

### Plant physiology

Overall, the appearance of both genotypes in each corresponding treatment was highly similar, and visually, no differential decline in vitality with increasing NaCl intensity was seen between them.

**Fig. 1:**
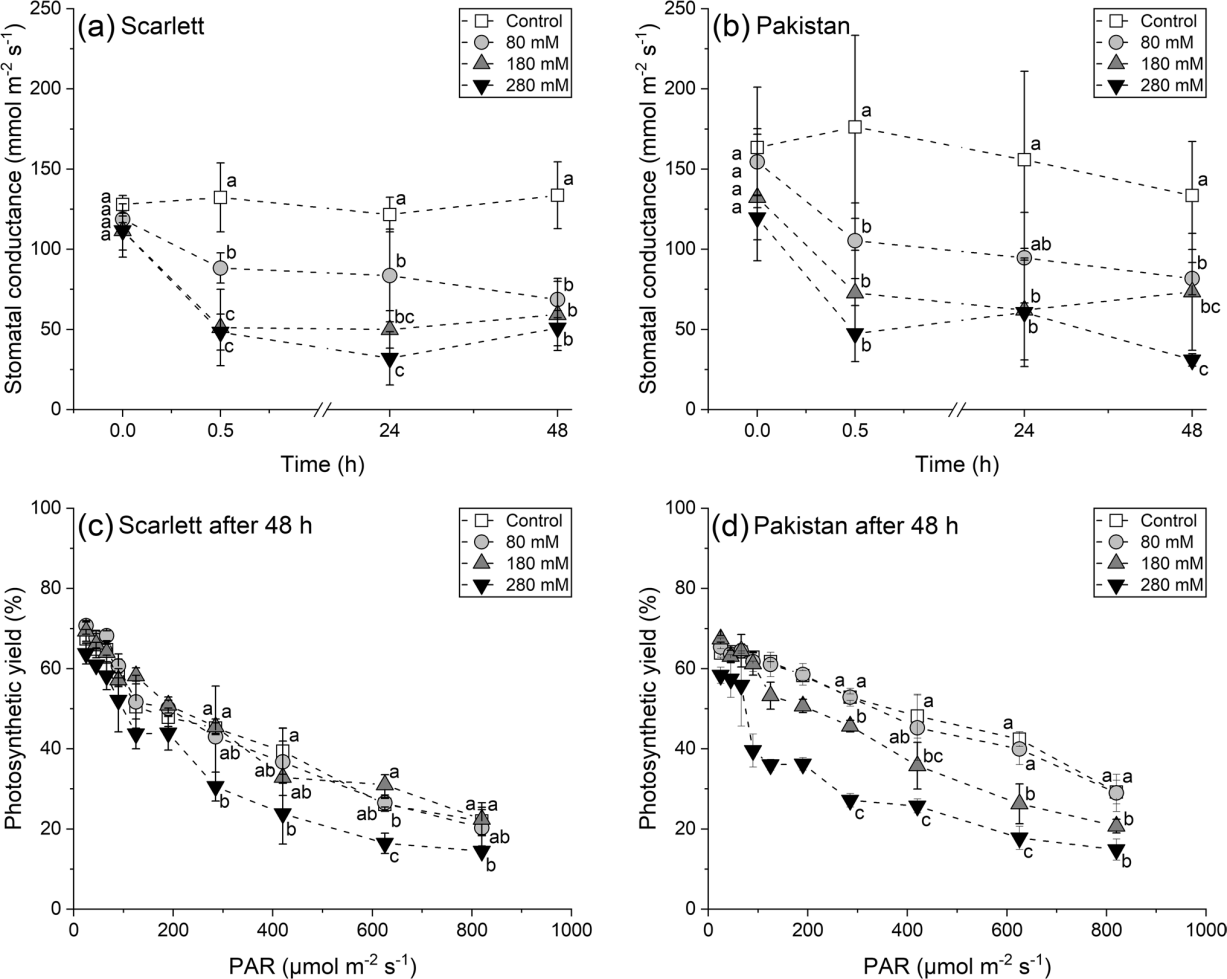
**a**–**d** Short-term physiological reactions of shoots of hydroponically cultivated barley plants subjected to different NaCl stress intensities. Stomatal conductance (**a**, **b**) and photosynthetic performance (**c**, **d**) of the first developed leaves were measured non-invasively within and right after the first 48 h of NaCl exposure, respectively. Means with standard deviations are shown. Differential letters indicate significant differences at *P* < 0.05; *n* = 3 replicates

Also, only subtle differences between both genotypes could be observed while monitoring the quickly occurring NaCl stress-induced effects on stomatal transpiration as well as photosynthetic performance (Fig. 1). While the stomatal conductances of leaves of all stress treatments declined already 0.5 h after NaCl application, but then basically plateaued for the remaining 48 h, Pakistan showed slightly higher values than Scarlett on some instances (Fig. 1a, b). These small differences were a little bit more pronounced for the photosynthetic yield challenged by light curves, in which Pakistan showed an overall slightly better performance with increasing light intensity than Scarlett, but was conversely also affected by the two higher NaCl concentrations of 180 and 280 mM more severely (Fig. 1c, d).

**Fig. 2:**
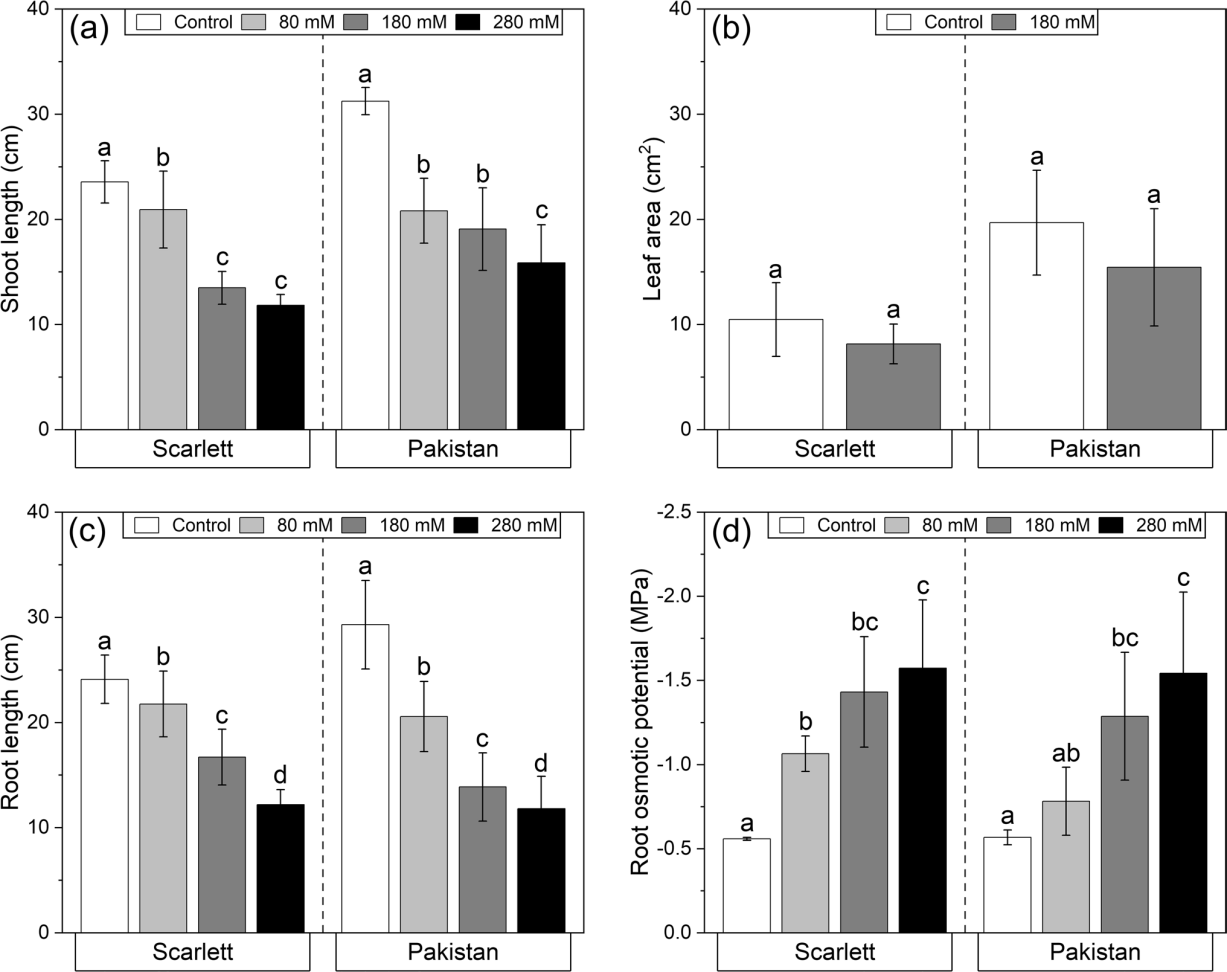
**a**–**d** Physiological parameters of shoots and roots of hydroponically cultivated barley plants subjected to different NaCl stress intensities. Shoot lengths (**a**), first leaf areas (**b**), seminal root lengths (**c**), and seminal root osmotic potentials (**d**) were estimated on the day of harvest after 12 days of cultivation, including 6 days of NaCl exposure. Means with standard deviations are shown. Differential letters indicate significant differences at *P* < 0.05; *n* = 7–21 (**a**), 3 (**b**), 26–52 (**c**), and 3 replicates (**d**)

After 6 days of salinity exposure, the shoot lengths of both genotypes were significantly reduced following the applied stress intensity, meaning that higher NaCl concentrations resulted in lower shoot elongations (Fig. 2a). Nonetheless, the surface areas of the first leaves of both genotypes cultivated in 180 mM NaCl were not significantly smaller than those of the control treatment (Fig. 2b). In accordance with the shoot lengths, also the seminal root lengths were shorter with increasing NaCl concentration, again similarly between both genotypes (Fig. 2c). This likewise reaction also extended to the osmotic potentials of roots, which for both genotypes comparably decreased with increasing NaCl intensity (Fig. 2d).

**Fig. 3:**
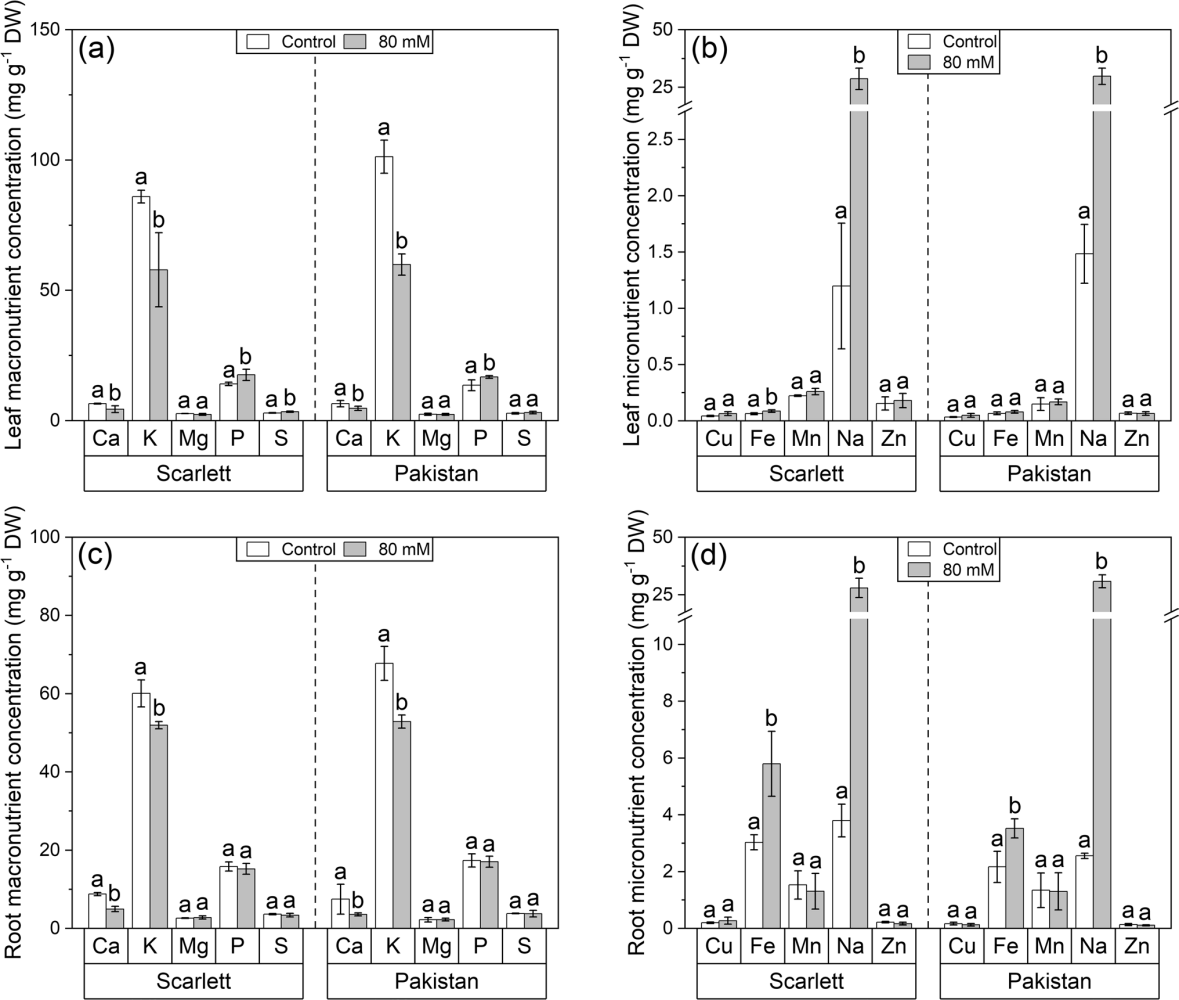
**a**–**d** Ionomes of leaves and roots of hydroponically cultivated barley plants subjected to the lowest tested NaCl stress intensity. This concentration was chosen, because previous data of this study had already indicated that 180 and 280 mM would reliably enforce reactions, but it was of greater interest to see if this was also already the case with the lowest NaCl stress treatment (80 mM) tested in this study. Macro- (**a**, **c**) and micronutrient (**b**, **d**) concentrations of the first leaves and seminal roots were measured after 12 days of cultivation, including 6 days of NaCl exposure. Means with standard deviations are shown. Differential letters indicate significant differences at *P* < 0.05; *n* = 3–6 replicates

Adaptations of the leaf and root macro- and micronutrient profiles were tested with the lowest NaCl intensity of 80 mM (Fig. 3). This concentration was chosen, because previous data of this study had already indicated that 180 and 280 mM would reliably enforce reactions, but it was of greater interest to see if this was also already the case with the lowest NaCl stress treatment (80 mM) tested in this study. In fact, both in leaves (Fig. 3a, b) and in seminal roots (Fig. 3c, d), it could be observed that sodium (Na) accumulated in strikingly high amounts, while calcium (Ca) and potassium (K) were significantly reduced in both genotypes. Interestingly, the 80 mM NaCl exposure led to slight increases in phosphorus (P) in leaves (Fig. 3a) and more pronounced increases in iron (Fe) in seminal roots (Fig. 3d) of both genotypes.

### Histochemical observation of apoplastic root barriers

**Fig. 4:**
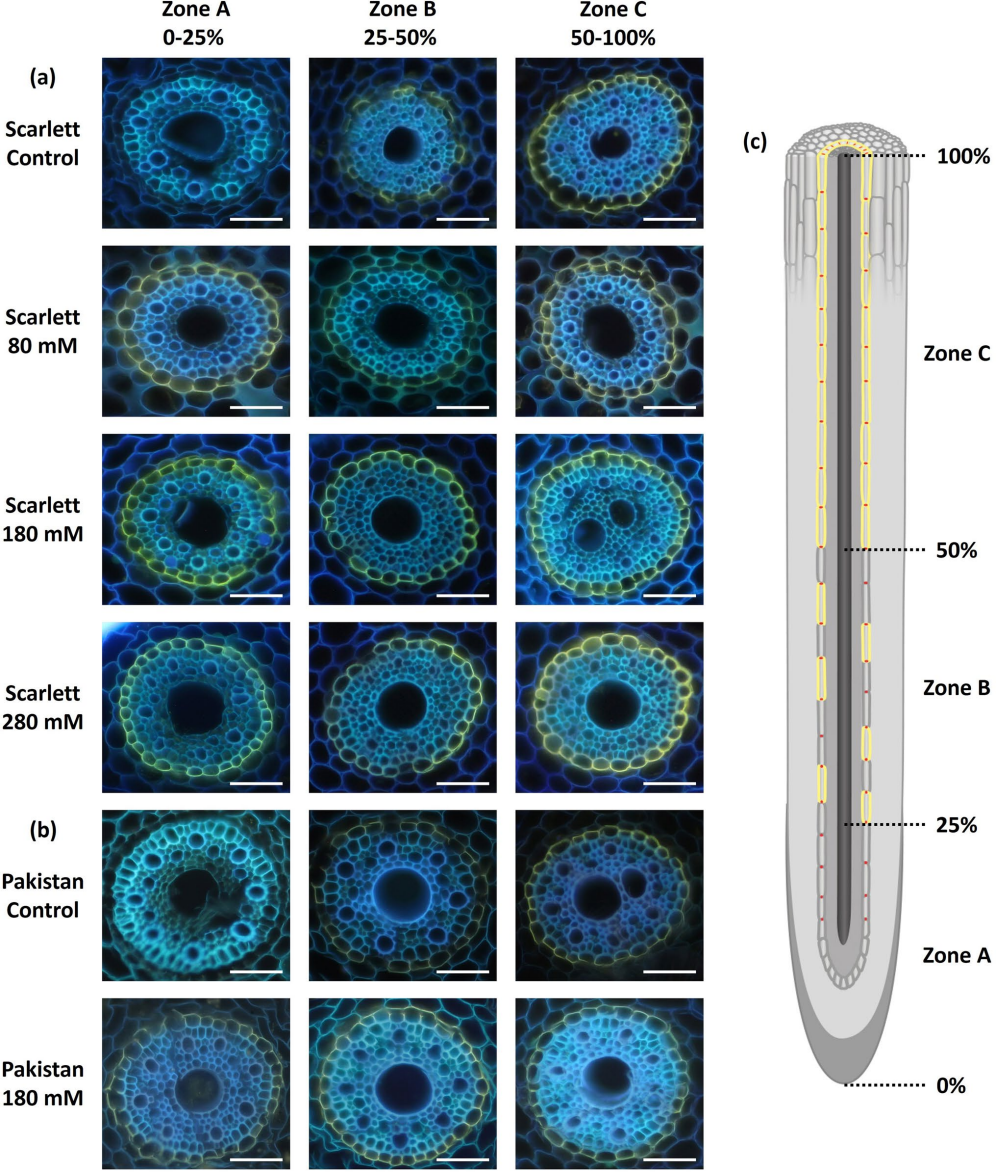
**a**–**c** Histochemical visualization of endodermal suberin lamellae development of seminal roots of hydroponically cultivated barley plants subjected to different NaCl stress intensities. The cultivar Scarlett (**a**) was analyzed for all NaCl concentrations, while the wild accession Pakistan (**b**) was screened histochemically only after exposure to the medium tested NaCl intensity. The analysis was performed after 12 days of cultivation, including 6 days of NaCl exposure. The seminal root cross-sections were stained with fluorol yellow 088, and representative photographs of the relative distances from the root tip (0%) to the root base (100%) are given. **c** Three functional zones of no suberization (Zone A, 0–25%), patchy suberization (Zone B, 25–50%), and full suberization (Zone C, 50–100%) were defined based on the endodermal suberin lamellae development observed in control conditions, and the schematic diagram is taken and adapted from Kreszies et al. (2019) and Grünhofer et al. (2021b); scale bars = 50 µm

Endodermal Casparian bands did not show any differences in development between the control and any of the treatments and were always already developed at around 12.5% relative root length before the onset of endodermal suberization (no data shown). The formation of endodermal suberin lamellae in reaction to NaCl stress exposure was most thoroughly investigated histochemically with seminal roots of Scarlett (Fig. 4a) and less extensive only for the medium intensity of 180 mM NaCl for Pakistan (Fig. 4b). The reductive modification in the screening procedure of Pakistan is justified by the presented uniform findings between both accessions and the fact that ultimately only the chemical analysis will deliver quantifiable results. In both accessions, it was clearly visible that the non-stress hydroponic control conditions resulted in the exact three functional developmental zones that were previously described under the same experimental conditions (Fig. 4c; Kreszies et al. 2019; Grünhofer et al. 2021b) and that already 80 mM NaCl provoked a considerable suberization of not only the patchy Zone B (25–50%) but even the previously non-suberized Zone A (0–25%). The two higher NaCl concentrations of 180 and 280 mM did not result in histochemically observable higher endodermal suberization of the seminal roots of Scarlett. Accordingly, the functional zones of seminal roots of the wild accession Pakistan (Fig. 4b) were developed very similar to those of Scarlett in control conditions, and also the root tip (Zone A, 0–25%) of Pakistan was almost fully suberized after treatment with the medium intensity of 180 mM NaCl. The formation of a suberized exodermis has never been observed in any NaCl treatment or genotype.

### Chemical analysis of cuticular wax, cutin, and suberin

The quality (composition of constituents) of the cuticular wax, cutin, and suberin of both Scarlett and Pakistan was as described in detail previously (Kreszies et al. 2019; Shellakkutti et al. 2022), which is why the data presentation in this study was primarily focused on overall quantities. If alterations in total amounts were observed, all functional groups and monomers were usually affected uniformly. Additional details about the exact monomeric composition are given in the corresponding Figs. S6-S11.

**Fig. 5:**
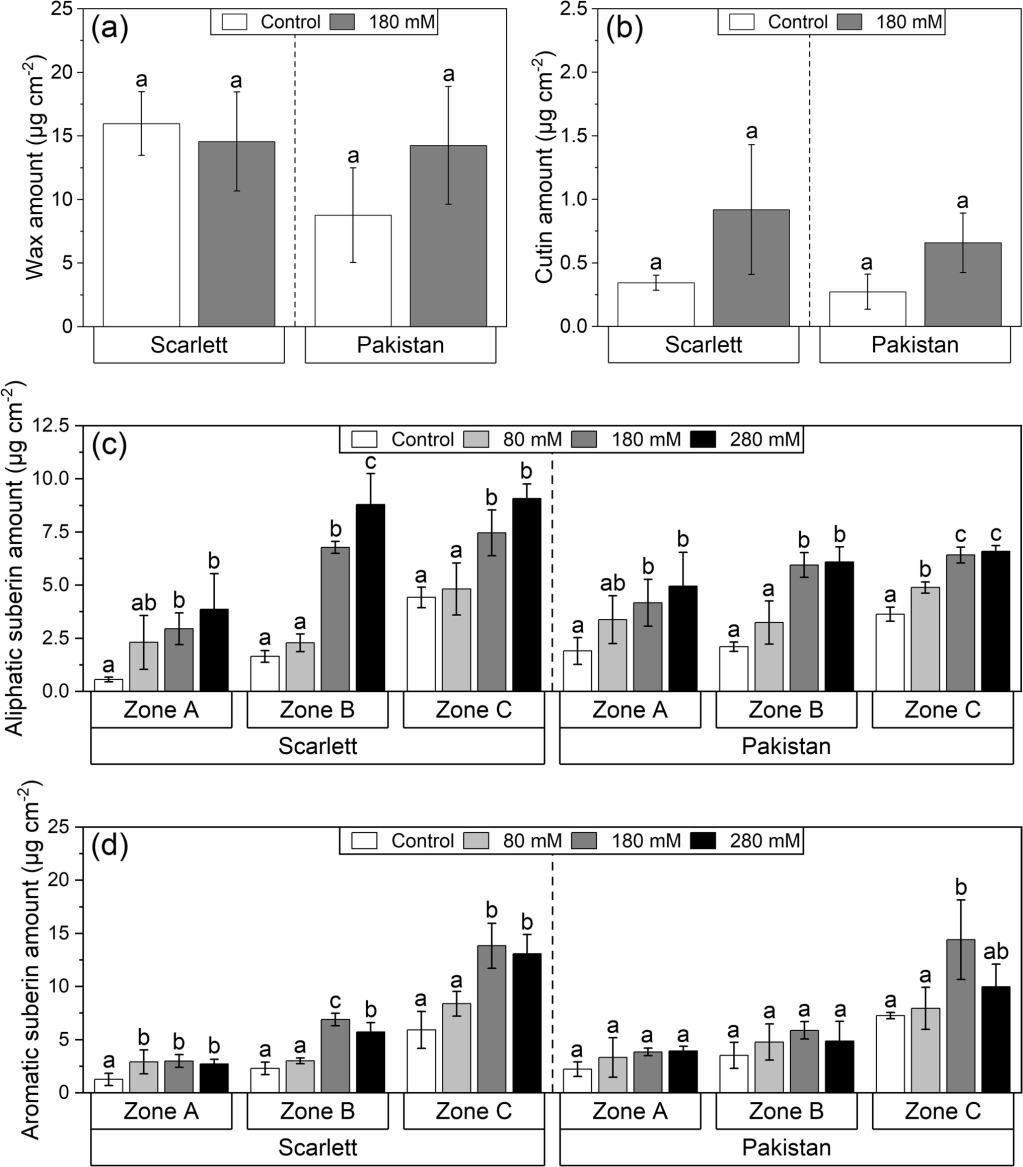
**a**–**d** Chemical analysis of shoot and root apoplastic barriers of hydroponically cultivated barley plants subjected to different NaCl stress intensities. First leaf cuticular wax (**a**) and cutin amounts (**b**) were measured for the medium tested NaCl intensity, while seminal root endodermal aliphatic (**c**) and aromatic suberin amounts (**d**) were investigated for all NaCl stress intensities. The analysis was performed after 12 days of cultivation, including 6 days of NaCl exposure. The seminal roots were divided into three functional root zones. Only overall amounts are shown here, and more details about monomeric composition are given in the corresponding Figs. S6-S11. Means with standard deviations are shown. Differential letters indicate significant differences at *P* < 0.05; *n* = 3 replicates

While, for leaves, the cuticular wax amounts of Scarlett did not change in response to 180 mM NaCl, there was a slight but nonetheless insignificant trend of increased amounts in Pakistan (Fig. 5a). This trend even extended into the cutin analysis of both genotypes, but also here the differences between the means were not significant (Fig. 5b).

In contrast, the endodermal suberin amounts of seminal roots were affected by the different NaCl intensities much more evidently (Fig. 5c, d). Here, sharp increases of aliphatic suberin could be observed in all root zones and for both genotypes, with the medium (180 mM) and high (280 mM) NaCl concentrations sticking out the most (Fig. 5c). Moreover, it appeared that in these two treatments, the aliphatic suberin amounts of the middle Zone B already matched those measured in Zone C in both genotypes. Although statistically not significant, strong increases of aliphatic suberin were seen primarily in Zone A after only 80 mM NaCl exposure, which fitted the previous observations in the histochemical analysis (Fig. 4). Aromatic suberin was overall less affected and significantly increased especially throughout all root zones of Scarlett rather than in Pakistan (Fig. 5d).

### Comparison of both genotypes

All of the presented data (Figs. 1, 2, 3, 4, 5) were additionally re-plotted and statistically analyzed to specifically investigate between-accession differences (Figs. S1-S5). However, truly meaningful biological differences were not evident to the authors and were thus not subsequently discussed in great detail.

## Discussion

According to Polle and Chen (2015), the three NaCl concentrations (80, 180, and 280 mM) investigated in this study would fall right between the category medium and high (80 mM), as well as into the category of extreme (180, 280 mM) salt stress for most plants. However, for barley, it has been shown that salt-resistant genotypes can withstand concentrations of up to 300 mM (Flowers and Hajibagheri 2001). The fact that both barley genotypes investigated here were able to withstand even the highest of these intensities substantiates barley’s standing as a comparably halophytic crop plant species (Ismail and Horie 2017). For short-term physiological reactions, it can be expected that they will not differ too dramatically from those observed after osmotic stress exposure, because both types of stress share the first initial osmotically driven phase of stress reactions, but salinity will unfold its full destructive potential in its unique second and slower ionic phase (Munns and Tester 2008). Thus, especially the long-term effects of NaCl exposure observed for both genotypes in this study will be benchmarked against those described for osmotic stress in hydroponics or water withdrawal in soil (Kreszies et al. 2020a; Shellakkutti et al. 2022; Suresh et al. 2024).

### Plant physiology

The quick reduction of stomatal conductivity even within the first hour of stress application (Fig. 1a, b) has been described before, also for barley (Fricke et al. 2004), and is not surprising, because stomatal closure is one of the plant's fastest options to react to encountered water status imbalances. These are caused by the inversion of the water potential gradient, which is usually directed from the rhizosphere into the root tissue (Grünhofer and Schreiber 2023), and stomatal closure quickly restricts the uncontrolled loss of water across the leaves (Kerstiens 1996). Similarly, the negative influence of NaCl on the photosynthetic machinery a few days after exposure (Fig. 1c, d) is well known and results from the reduction of CO_2_ uptake through stomata, ionic imbalances within the chloroplasts, and the oxidative damage caused to photosystems and membranes (Munns et al. 2020). A decreased photosynthetic yield, along with a reduced cell turgor pressure, limits osmotically driven cell expansion (Jaleel et al. 2009). Over time, this will translate into lower biomass accumulation and shorter cells, leading to reduced shoot and root lengths (Fig. 2a, c).

The fact that reliable data for leaves of all NaCl stress treatments (with a focus on 180 mM for leaves in this study; Fig. 2b) could only be gathered for the first leaf, in contrast to −0.8 MPa osmotic stress treatment where both leaf 1 and leaf 2 could be investigated (Shellakkutti et al. 2022), underpins the comparatively higher stress level caused by NaCl even if the same degree of osmotic stress (180 mM ≈ −0.8 MPa) is applied.

Nonetheless, the osmotic potentials of the roots (Fig. 2d) clearly indicate that both genotypes were very well capable of dealing with the NaCl treatments of all intensities, because the measured osmotic potential of the tissue is always just slightly more negative than the surrounding hydroponic medium (80 mM ≈ −0.4 MPa; 180 mM ≈ −0.8 MPa; 280 mM ≈ −1.2 MPa) and the roots were thus able to adapt to each condition adequately. This reactive decrease permits the maintenance of an osmotic potential gradient which is directed inwards, thus facilitating water uptake (Grünhofer and Schreiber 2023). In barley roots, this might be achieved by the accumulation of osmotically active substances such as proline (Muzammil et al. 2018) and the build-up of sodium (Na) (Fig. 3) in different plant tissues (Flowers and Hajibagheri 2001).

The mineral element analysis of leaves (Fig. 3a, b) and roots (Fig. 3c, d) revealed a significant uptake of Na^+^ ions into both organs. The circumstance that no difference in sodium concentration was found between both genotypes or between both organs suggests that in both the cultivated and wild barley, the majority of Na^+^ ions taken up by the roots were transported to the shoots without any discrimination even in the lowest NaCl intensity (80 mM) tested in this study. However, it must be recapitulated here that 80 mM NaCl marks the border between the category of a medium (40–80 mM) and a high (80–160 mM) salt stress intensity and is thus of great global agronomical relevance (Polle and Chen 2015). However, the observed lack of control of organ-specific sodium concentrations might not be as important for the halophytic barley in comparison to more salt-susceptible species (Munns et al. 2006), because leaf epidermal cells of barley have previously been shown to readily make use of inorganic solutes like cationic (such as Na^+^) and anionic (such as Cl^−^) osmolytes to generate osmolality during high-salt conditions (Fricke et al. 1994). As a result, the high concentrations of sodium are well known to disturb the uptake of other cationic nutrients, such as K^+^ or Ca^2+^ (Zörb et al. 2019), which has also been observed in both genotypes and both organs measured in this study (Fig. 3a, c). The measured higher Na^+^ and (consequently) lower K^+^ concentrations are mainly based on their direct competition due to the similar ion properties, which can easily cause ion imbalances. It is known for barley that, already after 1 h of 80 mM NaCl exposure, efflux of K^+^ from the roots may occur due to decreased membrane stability (Chen et al. 2005). Thus, stronger apoplastic barriers are needed to prevent the influx of toxic Na^+^ (see following ‘Apoplastic root barriers’ section) and efflux of K^+^ across the endodermis (Vestenaa et al. 2024). The fact that ionic imbalances can lead to a variety of physiological responses, for example, stomatal closure, decreased photosynthesis, and impaired protein biosynthesis, has been well documented for barley before (Zörb et al. 2019). What was more surprising was the significant increase of iron (Fe) in roots (Fig. 3b, d). This has, although rarely, been reported for roots in other species (Chavan and Karadge 1980; Turan et al. 2010), but to the best of the author’s knowledge, no clear mechanistic explanation has been described so far. Thus, it might only be speculated at this point that the differential degree of root suberization observed in this study (Figs. 4, 5c; further elaborated on later) might somehow be connected to the iron uptake dynamics of roots, as has previously been indicated by a study on suberin-deficient poplars (Grünhofer et al. 2024) and metal-deficient Arabidopsis (Barberon et al. 2016). Nevertheless, no reliable conclusion can be drawn here due to different iron acquisition strategies of monocotyledonous Poaceae, including barley, and dicotyledonous plants like poplar and Arabidopsis (Marschner and Römheld 1994).

### The leaf cuticle

Except for trends of increase, no significant differences in associated cuticular wax (Fig. 5a) and cutin (Fig. 5b) amounts could be identified for both genotypes. It is evident that these trends would be diminished even further once the absolute wax or cutin amount per leaf (in µg) is calculated by relating the amount per area (in µg cm^−2^) to the entire leaf surface areas (in cm^2^; being slightly smaller after stress exposure), which has been done previously for barley plants subjected to −0.8 MPa of osmotic stress (Shellakkutti et al. 2022). No change in cuticular wax amounts of barley leaves was also identified in an earlier study after 4–7 days of three different NaCl intensity (50, 100, and 150 mM) treatments (Even et al. 2018). In another short-term (2–3 days) salt exposure (100 mM) experiment, the rate of cuticular wax deposition was found to be reduced by similar proportions to the reduction in leaf elongation velocity, which also resulted in no change in cuticular wax amount per unit leaf surface area (Fricke et al. 2006). All three above-mentioned studies either speculated about or even measured both possible leaf water loss characteristics (stomatal and cuticular transpiration) and concordantly concluded that it is exclusively the stomatal regulation rather than cuticular permeance leading to declines in foliar dehydration (Fricke et al. 2006; Even et al. 2018; Shellakkutti et al. 2022). From a greater perspective, this is not too surprising, because even increased amounts of cuticular wax were shown not to restrict the cuticular transpiration in poplar (Grünhofer et al. 2022a) and it rather seems to be a minimum effective amount of cuticular wax in combination with stomatal closure that secures leaves from water loss to the highly water-deficient atmosphere (Grünhofer and Schreiber 2023).

### Apoplastic root barriers

For primary roots, the situation might be slightly different, because they represent organs designed to take up water from the soil and lack associated waxes (Schreiber et al. 1999) that are key components reducing water loss in cuticles (Schönherr 1976). Instead, they must orchestrate the uptake or exclusion of beneficial nutrients or toxic contaminants, respectively (Franke and Schreiber 2007). Both are achieved by the coordinated deposition of (endodermal or exodermal) Casparian bands and suberin lamellae in chronological order (Krömer 1903). Visual evidence of the former was not shown in this study, because the endodermal Casparian bands were found to consistently develop at around 12.5% relative root length and irrespective of the three applied NaCl (80, 180, and 280 mM) intensities, exactly as has been reported previously for three osmotically matched osmotic stress (− 0.4, − 0.8, and − 1.2 MPa) treatments (Kreszies et al. 2019). In contrast, the endodermal suberin lamellae deposition yielded remarkable differences. While in response to osmotic stress of all intensities, it was especially the patchy suberized functional root zone (Zone B, 25–50% relative root length) showing increased endodermal suberization after investigation by histochemical analysis (Kreszies et al. 2019), the exposure to salinity in this study resulted in an outstanding suberization of especially the otherwise non-suberized (Zone A, 0–25%) root tip (Fig. 4). In both studies, the morphologically observed adaptation could be confirmed by chemical analysis, which in the case of the more challenging salinity treatments revealed two additional interesting findings: (i) in both genotypes, the aliphatic suberin fraction of Zone B and Zone C of the 180 and 280 mM NaCl treatment approached a certain maximum threshold value of endodermal suberin deposition (Fig. 5c), which has already been identified for Scarlett (approx. 6–9 µg cm^−2^) before and might be sufficient to enable its full physiological potential (Grünhofer et al. 2021b); and (ii) especially in Scarlett, the aromatic suberin fraction exhibited a stronger increase (Fig. 5d) than had been observed in reaction to pure osmotic stress previously (Kreszies et al. 2019). However, since aromatic constituents are known to be highly abundant in cell walls of Poaceae species anyway (Carpita 1996), introducing the risk of overestimation, and because especially aliphatic rather than aromatic suberin is speculated to be affecting root transport properties (Hose et al. 2001; Ranathunge and Schreiber 2011), the following transport physiological considerations will focus on the aliphatic suberin fraction.

A preferential suberization of the root tip in response to NaCl exposure has been reported before in cotton (Reinhardt and Rost 1995), as well as in wheat and poplar where also a concomitant significant reduction in the osmotic (OS) and lesser effects on the hydrostatic (HY) hydraulic conductivity (Lp_r_) of roots, and thus the apoplastic contribution to overall water uptake, were identified (Grünhofer et al. 2022b; Lu and Fricke 2023). According to the composite transport model of roots (Ranathunge et al. 2017; Kim et al. 2018), water potential gradients are acting on the Lp_r_(OS) (reflecting the symplastic and transcellular pathway), while mainly hydrostatic pressure gradients and, to a lesser extent, osmotic forces are orchestrating the Lp_r_(HY) (comprising the symplastic, transcellular, and apoplastic pathway). In barley, it has been observed that a reduced water flow along the osmotically driven transcellular path after salt stress could be attributed primarily to the reduction in the activity of aquaporins rather than increased endodermal suberin deposition (Knipfer et al. 2021). This was slightly different after exposure to pure osmotic stress leading to higher suberization in Zone B, where the modern cultivar Scarlett and the wild accession Pakistan were found to exhibit no reductions in osmotic (in Scarlett and Pakistan) but, if anything, in hydrostatic (in Scarlett but not Pakistan) hydraulic conductivity (Kreszies et al. 2020a). Thus, rather than modulating (transcellular) water flow which is most quickly and effectively regulated by aquaporin gating (Steudle 2000), the elevated suberization of the endodermis of especially the root tip (Zone A) might contribute to the long-term shielding of the stele from the uptake of toxic NaCl (Munns and Tester 2008). The root endodermis is known as a checkpoint for nutrients (Barberon 2017), and particularly, the root tip has been described to be the most conductive root zone for ions (Foster and Miklavcic 2016). Here, increased levels of suberin would significantly reduce the membrane surface area exposed to NaCl (Enstone et al. 2003), and intermicrofibrillar spaces or plasmodesmata could be sealed (Meyer and Peterson 2013). However, especially in the case of barley, this hypothesis is challenged by the previous reports that, even with strong induction of endodermal suberization after salt stress, > 99% of the net amount of salt taken up by the barley plants studied was transported along the transcellular path (Lu and Fricke 2023). Thus, further and more detailed investigation in this regard will be needed in the future.

### Comparison of both genotypes

Barley itself already represents a comparably salt-tolerant crop species and will consequently be of importance for improving the salt tolerance of other food crops (Ismail and Horie 2017). However, additionally understanding which further traits could help to even enhance the resistance of different barley genotypes should not be of lesser value. Modern cultivars and wild accessions have long been known to exhibit pronounced genetic variability regarding salt tolerance (Jaradat et al. 2004; Zhu et al. 2015), but unraveling the exact physiological mechanisms is still a steadily ongoing process.

The detailed comparison of already acquired and newly generated data of Scarlett and Pakistan (Table 1) has shown that, overall, both genotypes suffered from all abiotic stress conditions in a similar manner. In regard to the chemistry and functionality of the leaf cuticle, the biosynthetic reaction of both genotypes was either very similar or Pakistan exhibited slightly higher amplitudes, which did not result in different functional outcomes. This was distinctly different for the deposition of apoplastic root barriers, because here the modern cultivar Scarlett was significantly more reactive than Pakistan, even during cultivation in soil, and deposited considerably more aliphatic endodermal suberin in response to all stress conditions.

The slightly better performance of Pakistan during pure osmotic stress, attributed to its wider genetic background and putatively accompanying more diverse coping mechanisms (Kreszies et al. 2020a), could not be supported by the findings of this study investigating an even more intense abiotic stress additionally encompassing a second ionic phase of stress chronology (Munns 2002). This might potentially be explained by the consistently observed lower degree of endodermal suberization in the roots of Pakistan. While its presumed threshold value of endodermal suberin deposition might be adequate to retain accumulated compatible solutes (such as the osmolyte proline) within the central cylinder, leading to a sufficient decline in root osmotic potential to cope with pure osmotic stress (osmotic phase), it might not be sufficient to effectively exclude toxic Na^+^ and Cl^−^ ions from being taken up during longer exposure times (ionic phase). Instead, this property could be established by the induction of a suberized exodermis, which has not been observed in either genotype in this study. The exodermis is a root trait well known for its salt protective qualities (Liu and Kreszies 2023) that has previously been observed, for example, in the modern cultivars Alexis (Gierth et al. 1999; Lehmann et al. 2000) and Jana (Reissinger et al. 2003) or the wild accession termed Jordan (Kreszies et al. 2020a).

We conclude that, in contrast to our expectations, hardly any biologically meaningful differences could be observed between both accessions in response to NaCl exposure in this study (Figs. S1-S5). However, it can be hypothesized that the repetition of these experiments with one or more of such exodermis-developing cultivated and wild barley genotypes would be a highly valuable approach to generate further insights into the functional role of an exodermis during salinity exposure. A deeper mechanistic understanding should result in more appropriately selected breeding targets or pave the way for purposeful biotechnological engineering.

## Supplementary Information

Below is the link to the electronic supplementary material.

Supplementary file1 (DOCX 3185 KB)

## Data Availability

Data that support the findings of this study are available from the corresponding author upon reasonable request.

## References

[CR1] Abramoff MD, Magalhães PJ, Ram SJ (2004) Image processing with imageJ. Biophotonics Int 11:36–42

[CR2] Armand T, Cullen M, Boiziot F, Li L, Fricke W (2019) Cortex cell hydraulic conductivity, endodermal apoplastic barriers and root hydraulics change in barley (*Hordeum vulgare* L.) in response to a low supply of N and P. Ann Bot 124:1091–1107. 10.1093/aob/mcz113 31309230 10.1093/aob/mcz113 PMC7145705

[CR3] Baales J, Zeisler-Diehl VV, Schreiber L (2021) Analysis of extracellular cell wall lipids: wax, cutin, and suberin in leaves, roots, fruits, and seeds. In: Bartels D, Dörmann P (eds) Plant lipids: methods and protocols, 1st edn. Springer, New York, pp 275–293. 10.1007/978-1-0716-1362-7_15 10.1007/978-1-0716-1362-7_15 34047982

[CR4] Barberon M (2017) The endodermis as a checkpoint for nutrients. New Phytol 213:1604–1610. 10.1111/nph.14140 27551946 10.1111/nph.14140

[CR5] Barberon M, Vermeer JEM, De Bellis D, Wang P, Naseer S, Andersen TG, Humbel BM, Nawrath C, Takano J, Salt DE (2016) Adaptation of root function by nutrient-induced plasticity of endodermal differentiation. Cell 164:447–459. 10.1016/j.cell.2015.12.021 26777403 10.1016/j.cell.2015.12.021

[CR6] Brundrett MC, Enstone DE, Peterson CA (1988) A berberine-aniline blue fluorescent staining procedure for suberin, lignin, and callose in plant tissue. Protoplasma 146:133–142. 10.1007/BF01405922

[CR7] Brundrett MC, Kendrick B, Peterson CA (1991) Efficient lipid staining in plant material with Sudan Red 7B or Fluoral Yellow 088 in polyethylene glycol-glycerol. Biotech Histochem 66:111–116. 10.3109/10520299109110562 1716161 10.3109/10520299109110562

[CR8] Carpita NC (1996) Structure and biogenesis of the cell walls of grasses. Annu Rev Plant Biol 47:445–476. 10.1146/annurev.arplant.47.1.445 10.1146/annurev.arplant.47.1.445 15012297

[CR9] Challinor AJ, Watson J, Lobell DB, Howden SM, Smith DR, Chhetri N (2014) A meta-analysis of crop yield under climate change and adaptation. Nat Clim Change 4:287–291. 10.1038/nclimate2153

[CR10] Chavan PD, Karadge BA (1980) Influence of salinity on mineral nutrition of peanut (*Arachis hypogea* L.). Plant Soil 54:5–13. 10.1007/BF02181995

[CR11] Chen Z, Newman I, Zhou M, Mendham N, Zhang G, Shabala S (2005) Screening plants for salt tolerance by measuring K^+^ flux: a case study for barley. Plant Cell Environ 28:1230–1246. 10.1111/j.1365-3040.2005.01364.x

[CR12] Coffey O, Bonfield R, Corre F, Althea Sirigiri J, Meng D, Fricke W (2018) Root and cell hydraulic conductivity, apoplastic barriers and aquaporin gene expression in barley (*Hordeum vulgare* L.) grown with low supply of potassium. Ann Bot 122:1131–1141. 10.1093/aob/mcy110 29961877 10.1093/aob/mcy110 PMC6324746

[CR13] Ellis RP, Forster BP, Robinson D, Handley LL, Gordon DC, Russell JR, Powell W (2000) Wild barley: a source of genes for crop improvement in the 21st century? J Exp Bot 51:9–17. 10.1093/jexbot/51.342.9 10938791

[CR14] Enstone DE, Peterson CA, Ma F (2003) Root endodermis and exodermis: structure, function, and responses to the environment. J Plant Growth Regul 21:335–351. 10.1007/s00344-003-0002-2

[CR15] Eshel A, Beeckman T (eds) (2013) Plant roots: the hidden half, 4th edn. CRC Press, New York

[CR16] Even M, Sabo M, Meng D, Kreszies T, Schreiber L, Fricke W (2018) Night-time transpiration in barley (*Hordeum vulgare*) facilitates respiratory carbon dioxide release and is regulated during salt stress. Ann Bot 122:569–582. 10.1093/aob/mcy084 29850772 10.1093/aob/mcy084 PMC6153476

[CR17] FAO (2024) Global status of salt-affected soils—main report. 10.4060/cd3044en

[CR18] Flowers TJ, Hajibagheri MA (2001) Salinity tolerance in Hordeum vulgare: ion concentrations in root cells of cultivars differing in salt tolerance. Plant Soil 231:1–9. 10.1023/A:1010372213938

[CR19] Foster KJ, Miklavcic SJ (2016) Modeling root zone effects on preferred pathways for the passive transport of ions and water in plant roots. Front Plant Sci 7:914. 10.3389/fpls.2016.00914 27446144 10.3389/fpls.2016.00914 PMC4917552

[CR20] Franke R, Schreiber L (2007) Suberin—a biopolyester forming apoplastic plant interfaces. Curr Opin Plant Biol 10:252–259. 10.1016/j.pbi.2007.04.004 17434790 10.1016/j.pbi.2007.04.004

[CR21] Fricke W, Leigh RA, Tomos AD (1994) Concentrations of inorganic and organic solutes in extracts from individual epidermal, mesophyll and bundle-sheath cells of barley leaves. Planta 192:310–316. 10.1007/BF00198565

[CR22] Fricke W, Akhiyarova G, Veselov D, Kudoyarova G (2004) Rapid and tissue-specific changes in ABA and in growth rate in response to salinity in barley leaves. J Exp Bot 55:1115–1123. 10.1093/jxb/erh117 15047763 10.1093/jxb/erh117

[CR23] Fricke W, Akhiyarova G, Wei W, Alexandersson E, Miller A, Kjellbom PO, Richardson A, Wojciechowski T, Schreiber L, Veselov D, Kudoyarova G, Volkov V (2006) The short-term growth response to salt of the developing barley leaf. J Exp Bot 57:1079–1095. 10.1093/jxb/erj095 16513814 10.1093/jxb/erj095

[CR24] Garthwaite AJ, von Bothmer R, Colmer TD (2005) Salt tolerance in wild *Hordeum* species is associated with restricted entry of Na^+^ and Cl^-^ into the shoots. J Exp Bot 56:2365–2378. 10.1093/jxb/eri229 16014366 10.1093/jxb/eri229

[CR25] Gierth M, Stelzer R, Lehmann H (1999) An analytical microscopical study on the role of the exodermis in apoplastic Rb^+^(K^+^) transport in barley roots. Plant Soil 207:209–218. 10.1023/A:1004437516331

[CR26] Grünhofer P, Schreiber L (2023) Cutinized and suberized barriers in leaves and roots: similarities and differences. J Plant Physiol 282:153921. 10.1016/j.jplph.2023.153921 36780757 10.1016/j.jplph.2023.153921

[CR27] Grünhofer P, Guo Y, Li R, Lin J, Schreiber L (2021a) Hydroponic cultivation conditions allowing the reproducible investigation of poplar root suberization and water transport. Plant Methods 17:129. 10.1186/s13007-021-00831-5 34911563 10.1186/s13007-021-00831-5 PMC8672600

[CR28] Grünhofer P, Schreiber L, Kreszies T (2021) Suberin in monocotyledonous crop plants: structure and function in response to abiotic stresses. In: Baluška F, Mukherjee S (eds) Rhizobiology: molecular physiology of plant roots. Springer Nature, Cham, pp 333–378. 10.1007/978-3-030-84985-6_19

[CR29] Grünhofer P, Herzig L, Sent S, Zeisler-Diehl VV, Schreiber L (2022a) Increased cuticular wax deposition does not change residual foliar transpiration. Plant Cell Environ 45:1157–1171. 10.1111/pce.14274 35102563 10.1111/pce.14274

[CR30] Grünhofer P, Stöcker T, Guo Y, Li R, Lin J, Ranathunge K, Schoof H, Schreiber L (2022b) *Populus* × *canescens* root suberization in reaction to osmotic and salt stress is limited to the developing younger root tip region. Physiol Plant 174:e13765. 10.1111/ppl.13765 36281836 10.1111/ppl.13765

[CR31] Grünhofer P, Heimerich I, Pohl S, Oertel M, Meng H, Zi L, Lucignano K, Bokhari SNH, Guo Y, Li R, Lin J, Fladung M, Kreszies T, Stöcker T, Schoof H, Schreiber L (2024) Suberin deficiency and its effect on the transport physiology of young poplar roots. New Phytol 242:137–153. 10.1111/nph.19588 38366280 10.1111/nph.19588

[CR32] Gunasekera D, Santakumari M, Glinka Z, Berkowitz GA (1994) Wild and cultivated barley genotypes demonstrate varying ability to acclimate to plant water deficits. Plant Sci 99:125–134. 10.1016/0168-9452(94)90169-4

[CR33] Haas M, Schreiber M, Mascher M (2019) Domestication and crop evolution of wheat and barley: genes, genomics, and future directions. J Integr Plant Biol 61:204–225. 10.1111/jipb.12737 30414305 10.1111/jipb.12737

[CR34] Harlan JR, Zohary D (1966) Distribution of wild wheats and barley. Science 153:1074–1080. 10.1126/science.153.3740.1074 17737582 10.1126/science.153.3740.1074

[CR35] Hen-Avivi S, Savin O, Racovita RC, Lee W-S, Adamski NM, Malitsky S, Almekias-Siegl E, Levy M, Vautrin S, Bergès H, Friedlander G, Kartvelishvily E, Ben-Zvi G, Alkan N, Uauy C, Kanyuka K, Jetter R, Distelfeld A, Aharoni A (2016) A metabolic gene cluster in the wheat *W1* and the barley *Cer*-*cqu* loci determines β-diketone biosynthesis and glaucousness. Plant Cell 28:1440–1460. 10.1105/tpc.16.00197 27225753 10.1105/tpc.16.00197 PMC4944414

[CR36] Herzig L, Uellendahl K, Malkowsky Y, Schreiber L, Grünhofer P (2025) In a different light: irradiation-induced cuticular wax accumulation fails to reduce cuticular transpiration. Plant Cell Environ 48:3632–3646. 10.1111/pce.15376 39806923 10.1111/pce.15376 PMC11963476

[CR37] Hoagland DR, Arnon DI (1950) The water-culture method for growing plants without soil. Circular. 347

[CR38] Hose E, Clarkson DT, Steudle E, Schreiber L, Hartung W (2001) The exodermis: a variable apoplastic barrier. J Exp Bot 52:2245–2264. 10.1093/jexbot/52.365.2245 11709575 10.1093/jexbot/52.365.2245

[CR39] Ismail AM, Horie T (2017) Genomics, physiology, and molecular breeding approaches for improving salt tolerance. Annu Rev Plant Biol 68:405–434. 10.1146/annurev-arplant-042916-040936 28226230 10.1146/annurev-arplant-042916-040936

[CR40] Jaleel CA, Manivannan P, Wahid A, Farooq M, Al-Juburi HJ, Somasundaram R, Panneerselvam R (2009) Drought stress in plants: a review on morphological characteristics and pigments composition. Int J Agric Biol 11:100–105

[CR41] Jaradat AA, Shahid M, Al-Maskri A (2004) Genetic diversity in the Batini barley landrace from Oman: II. Response to salinity stress. Crop Sci 44:997–1007. 10.2135/cropsci2004.9970

[CR42] Kerstiens G (1996) Cuticular water permeability and its physiological significance. J Exp Bot 47:1813–1832. 10.1093/jxb/47.12.1813

[CR43] Kim YX, Ranathunge K, Lee S, Lee Y, Lee D, Sung J (2018) Composite transport model and water and solute transport across plant roots: an update. Front Plant Sci 9:1–9. 10.3389/fpls.2018.00193 29503659 10.3389/fpls.2018.00193 PMC5820301

[CR44] Knipfer T, Danjou M, Vionne C, Fricke W (2021) Salt stress reduces root water uptake in barley (*Hordeum vulgare* L.) through modification of the transcellular transport path. Plant Cell Environ 44:458–475. 10.1111/pce.13936 33140852 10.1111/pce.13936

[CR45] Kotula L, Schreiber L, Colmer TD, Nakazono M (2017) Anatomical and biochemical characterisation of a barrier to radial O_2_ loss in adventitious roots of two contrasting *Hordeum marinum* accessions. Funct Plant Biol 44:845–857. 10.1071/FP16327 32480613 10.1071/FP16327

[CR46] Kreszies T, Shellakkutti N, Osthoff A, Yu P, Baldauf JA, Zeisler-Diehl VV, Ranathunge K, Hochholdinger F, Schreiber L (2019) Osmotic stress enhances suberization of apoplastic barriers in barley seminal roots: Analysis of chemical, transcriptomic and physiological responses. New Phytol 221:180–194. 10.1111/nph.15351 30055115 10.1111/nph.15351 PMC6586163

[CR47] Kreszies T, Eggels S, Kreszies V, Osthoff A, Shellakkutti N, Baldauf JA, Zeisler-Diehl VV, Hochholdinger F, Ranathunge K, Schreiber L (2020a) Seminal roots of wild and cultivated barley differentially respond to osmotic stress in gene expression, suberization, and hydraulic conductivity. Plant Cell Environ 43:344–357. 10.1111/pce.13675 31762057 10.1111/pce.13675

[CR48] Kreszies T, Kreszies V, Ly F, Thangamani PD, Shellakkutti N, Schreiber L (2020b) Suberized transport barriers in plant roots: The effect of silicon. J Exp Bot 71:6799–6806. 10.1093/jxb/eraa203 32333766 10.1093/jxb/eraa203

[CR49] Krömer K (1903) Wurzelhaut, hypodermis und endodermis der angiospermenwurzel. Biblioth Bot 59:1–160

[CR50] Kunst L, Samuels AL (2003) Biosynthesis and secretion of plant cuticular wax. Prog Lipid Res 42:51–80. 10.1016/S0163-7827(02)00045-0 12467640 10.1016/s0163-7827(02)00045-0

[CR51] Lehmann H, Stelzer R, Holzamer S, Kunz U, Gierth M (2000) Analytical electron microscopical investigations on the apoplastic pathways of lanthanum transport in barley roots. Planta 211:816–822. 10.1007/s004250000346 11144266 10.1007/s004250000346

[CR52] Liu T, Kreszies T (2023) The exodermis: a forgotten but promising apoplastic barrier. J Plant Physiol 290:154118. 10.1016/j.jplph.2023.154118 37871477 10.1016/j.jplph.2023.154118

[CR53] Lu Y, Fricke W (2023) Diurnal changes in apoplast bypass flow of water and ions in salt-stressed wheat (*Triticum**aestivum* L.) and barley (*Hordeum**vulgare* L.). Physiol Plant 175:e13955. 10.1111/ppl.13955 37323067 10.1111/ppl.13955

[CR54] Marschner H, Römheld V (1994) Strategies of plants for acquisition of iron. Plant Soil 165:261–274. 10.1007/BF00008069

[CR55] Mayer KFX, Waugh R, Brown JWS, Schulman A, Langridge P, Platzer M, Fincher GB, Muehlbauer GJ, Sato K, Close TJ, Wise RP, Stein N (2012) A physical, genetic and functional sequence assembly of the barley genome. Nature 491:711–716. 10.1038/nature11543 23075845 10.1038/nature11543

[CR56] Melino VJ, Plett DC, Bendre P, Thomsen HC, Zeisler-Diehl VV, Schreiber L, Kronzucker HJ (2021) Nitrogen depletion enhances endodermal suberization without restricting transporter-mediated root NO_3_^-^ influx. J Plant Physiol 257:153334. 10.1016/j.jplph.2020.153334 33373827 10.1016/j.jplph.2020.153334

[CR57] Meyer CJ, Peterson CA (2013) Structure and function of three suberized cell layers: epidermis, exodermis, and endodermis. In: Eshel A, Beeckman T (eds) Plant roots: the hidden half, 4th edn. CRC Press, New York, p 5.1-5.20

[CR58] Müller Y, Patwari P, Stöcker T, Zeisler-Diehl V, Steiner U, Campoli C, Grewe L, Kuczkowska M, Dierig MM, Jose S, Hetherington AM, Acosta IF, Schoof H, Schreiber L, Dörmann P (2023) Isolation and characterization of the gene *HvFAR1* encoding acyl-CoA reductase from the *cer-za.227* mutant of barley (*Hordeum vulgare*) and analysis of the cuticular barrier functions. New Phytol 239:1903–1918. 10.1111/nph.19063 37349864 10.1111/nph.19063

[CR59] Munns R (2002) Comparative physiology of salt and water stress. Plant Cell Environ 25:239–250. 10.1046/j.0016-8025.2001.00808.x 11841667 10.1046/j.0016-8025.2001.00808.x

[CR60] Munns R, Tester M (2008) Mechanisms of salinity tolerance. Annu Rev Plant Biol 59:651–681. 10.1146/annurev.arplant.59.032607.092911 18444910 10.1146/annurev.arplant.59.032607.092911

[CR61] Munns R, James RA, Läuchli A (2006) Approaches to increasing the salt tolerance of wheat and other cereals. J Exp Bot 57:1025–1043. 10.1093/jxb/erj100 16510517 10.1093/jxb/erj100

[CR62] Munns R, Day DA, Fricke W, Watt M, Arsova B, Barkla BJ, Bose J, Byrt CS, Chen Z-H, Foster KJ, Gilliham M, Henderson SW, Jenkins CLD, Kronzucker HJ, Miklavcic SJ, Plett D, Roy SJ, Shabala S, Shelden MC, Soole KL, Taylor NL, Tester M, Wege S, Wegner LH, Tyerman SD (2020) Energy costs of salt tolerance in crop plants. New Phytol 225:1072–1090. 10.1111/nph.15864 31004496 10.1111/nph.15864

[CR63] Muzammil S, Shrestha A, Dadshani S, Pillen K, Siddique S, Léon J, Naz AA (2018) An ancestral allele of *pyrroline-5-carboxylate synthase1* promotes proline accumulation and drought adaptation in cultivated barley. Plant Physiol 178:771–782. 10.1104/pp.18.00169 30131422 10.1104/pp.18.00169 PMC6181029

[CR64] Panta S, Flowers T, Lane P, Doyle R, Haros G, Shabala S (2014) Halophyte agriculture: success stories. Environ Exp Bot 107:71–83. 10.1016/j.envexpbot.2014.05.006

[CR65] Polle A, Chen S (2015) On the salty side of life: molecular, physiological and anatomical adaptation and acclimation of trees to extreme habitats. Plant Cell Environ 38:1794–1816. 10.1111/pce.12440 25159181 10.1111/pce.12440

[CR66] Ranathunge K, Schreiber L (2011) Water and solute permeabilities of *Arabidopsis* roots in relation to the amount and composition of aliphatic suberin. J Exp Bot 62:1961–1974. 10.1093/jxb/erq389 21421706 10.1093/jxb/erq389 PMC3060681

[CR67] Ranathunge K, Kim YX, Wassmann F, Kreszies T, Zeisler V, Schreiber L (2017) The composite water and solute transport of barley (*Hordeum vulgare*) roots: effect of suberized barriers. Ann Bot 119:629–643. 10.1093/aob/mcw252 28065927 10.1093/aob/mcw252 PMC5604597

[CR68] Reinhardt DH, Rost TL (1995) Salinity accelerates endodermal development and induces an exodermis in cotton seedling roots. Environ Exp Bot 35:563–574. 10.1016/0098-8472(95)00015-1

[CR69] Reissinger A, Winter S, Steckelbroeck S, Hartung W, Sikora RA (2003) Infection of barley roots by *Chaetomium globosum*: evidence for a protective role of the exodermis. Mycol Res 107:1094–1102. 10.1017/S0953756203008189 14563137 10.1017/s0953756203008189

[CR70] Richardson A, Franke R, Kerstiens G, Jarvis M, Schreiber L, Fricke W (2005) Cuticular wax deposition in growing barley (*Hordeum vulgare*) leaves commences in relation to the point of emergence of epidermal cells from the sheaths of older leaves. Planta 222:472–483. 10.1007/s00425-005-1552-2 15940461 10.1007/s00425-005-1552-2

[CR71] Schönherr J (1976) Water permeability of isolated cuticular membranes: The effect of pH and cations on diffusion, hydrodynamic permeability and size of polar pores in the cutin matrix. Planta 128:113–126. 10.1007/BF00390312 24430686 10.1007/BF00390312

[CR72] Schreiber L, Hartmann K, Skrabs M, Zeier J (1999) Apoplastic barriers in roots: chemical composition of endodermal and hypodermal cell walls. J Exp Bot 50:1267–1280. 10.1093/jxb/50.337.1267

[CR73] Shellakkutti N, Thangamani PD, Suresh K, Baales J, Zeisler-Diehl V, Klaus A, Hochholdinger F, Schreiber L, Kreszies T (2022) Cuticular transpiration is not affected by enhanced wax and cutin amounts in response to osmotic stress in barley. Physiol Plant 174:e13735. 10.1111/ppl.13735 35716005 10.1111/ppl.13735

[CR74] Steudle E (2000) Water uptake by plant roots: an integration of views. Plant Soil 226:45–56. 10.1023/A:1026439226716

[CR75] Suresh K, Bhattacharyya S, Carvajal J, Ghosh R, Zeisler-Diehl VV, Böckem V, Nagel KA, Wojciechowski T, Schreiber L (2024) Effects of water stress on apoplastic barrier formation in soil grown roots differ from hydroponically grown roots: histochemical, biochemical and molecular evidence. Plant Cell Environ 47:4917–4931. 10.1111/pce.15067 39110071 10.1111/pce.15067

[CR76] Szabolcs I (1989) Salt-affected soils. CRC Press, Boca Raton

[CR77] Turan MA, Elkarim AHA, Taban N, Taban S (2010) Effect of salt stress on growth and ion distribution and accumulation in shoot and root of maize plant. Afr J Agric Res 5:584–588. 10.5897/AJAR09.677

[CR78] Vestenaa MW, Husted S, Minutello F, Persson DP (2024) Endodermal suberin restricts root leakage of cesium: a suitable tracer for potassium. Physiol Plant 176:e14393. 10.1111/ppl.14393 38923555 10.1111/ppl.14393

[CR79] Xie W, Xiong W, Pan J, Ali T, Cui Q, Guan D, Meng J, Mueller ND, Lin E, Davis SJ (2018) Decreases in global beer supply due to extreme drought and heat. Nature Plants 4:964–973. 10.1038/s41477-018-0263-1 30323183 10.1038/s41477-018-0263-1

[CR80] Zhu M, Zhou M, Shabala L, Shabala S (2015) Linking osmotic adjustment and stomatal characteristics with salinity stress tolerance in contrasting barley accessions. Funct Plant Biol 42:252–263. 10.1071/FP14209 32480671 10.1071/FP14209

[CR81] Zörb C, Geilfus C-M, Dietz K-J (2019) Salinity and crop yield. Plant Biol 21:31–38. 10.1111/plb.12884 30059606 10.1111/plb.12884

