# Supplementary material for: Sodium chloride enhances suberization in seminal roots but does not affect cutinized leaf barriers in cultivated and wild barley
Source: Planta. 2025 Jun 15;262(2):28. doi: 10.1007/s00425-025-04743-9 (PMC12167721; doi:10.1007/s00425-025-04743-9)
Supplement: Supplementary file 1 — Supplementary file1 (DOCX 3185 KB) [file 425_2025_4743_MOESM1_ESM.docx]

**Sodium chloride enhances suberization in seminal roots but does not affect cutinized leaf barriers in cultivated and wild barley**

Paul Grünhofer^1,+,*^, Priya Dharshini Thangamani^1,2,+^, Lukas Schreiber^1^, Tino Kreszies^3,*^

Contact information:

^1^Department of Ecophysiology, Institute of Cellular and Molecular Botany, University of Bonn, Kirschallee 1, 53115 Bonn, Germany

^2^Department of Plant Cell Biology, Botanical Institute and Botanical Garden, University of Kiel, Am Botanischen Garten 5, 24118 Kiel, Germany

^3^Centre for Crop Systems Analysis, Wageningen University and Research, the Netherlands

^+^Shared first author, contributed equally to this work

^*^Author for correspondence: Paul Grünhofer

E-Mail: p.gruenhofer@uni-bonn.de

**Acknowledgements**

Financial support by the Deutsche Forschungsgemeinschaft (DFG, German Research Foundation; SCHR21/1; Project number 511193270) is highly appreciated. The authors thank Susanne Koch and Kirsten Fladung for technical assistance with the mineral nutrient analysis.


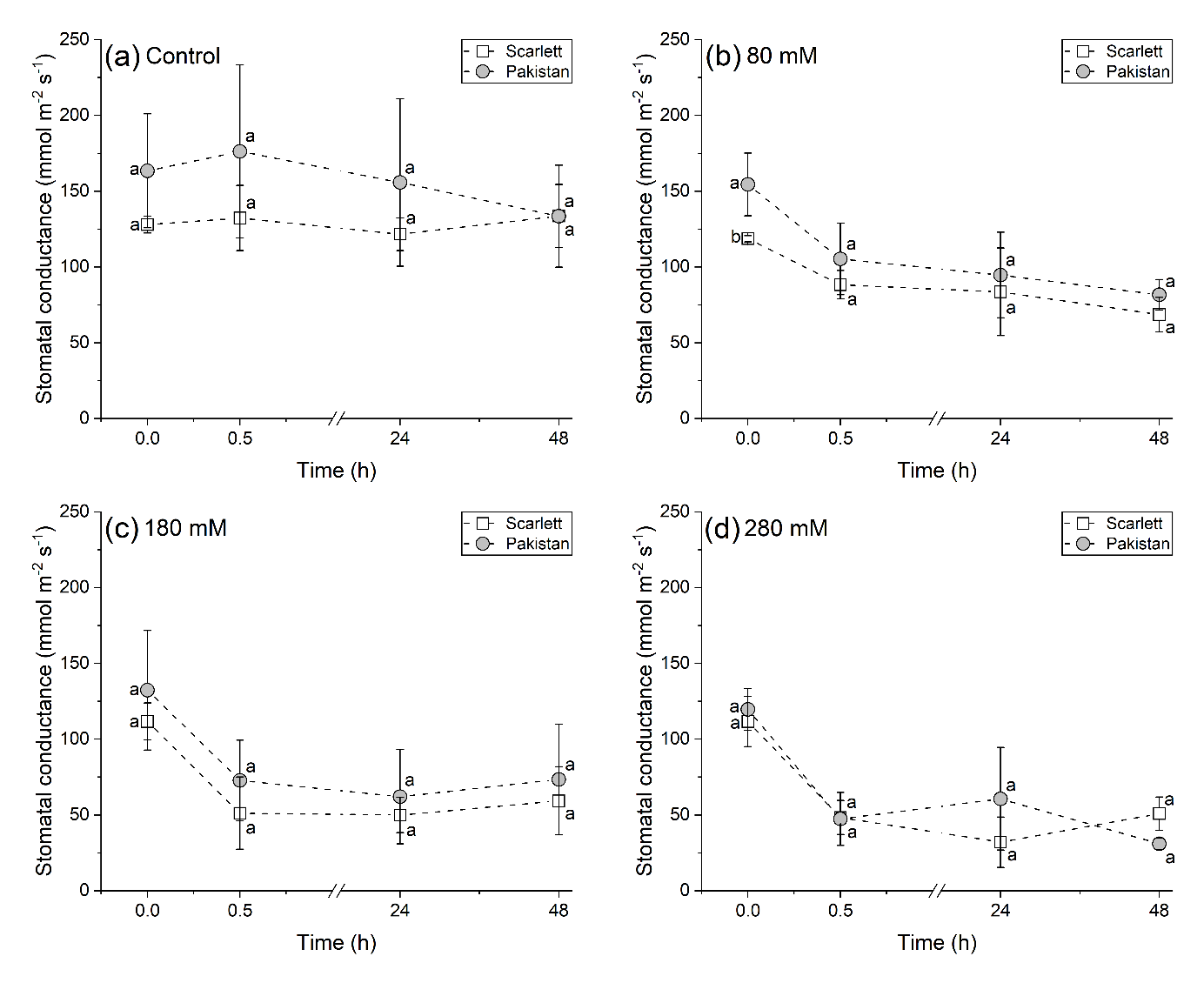


# Fig. S1a-d Short-term physiological reactions of shoots of hydroponically cultivated barley plants subjected to different NaCl stress intensities. The data is identical to Fig. 1a and 1b but plotted differently to visualize potential differences between the accessions. Stomatal conductance of the first developed leaves was measured non-invasively within the first 48 hours of control treatment (a), as well as 80 (b), 180 (c), and 280 mM NaCl (d) exposure. Means with standard deviations are shown. Differential letters indicate significant differences at *P* < 0.05; *n* = 3 replicates


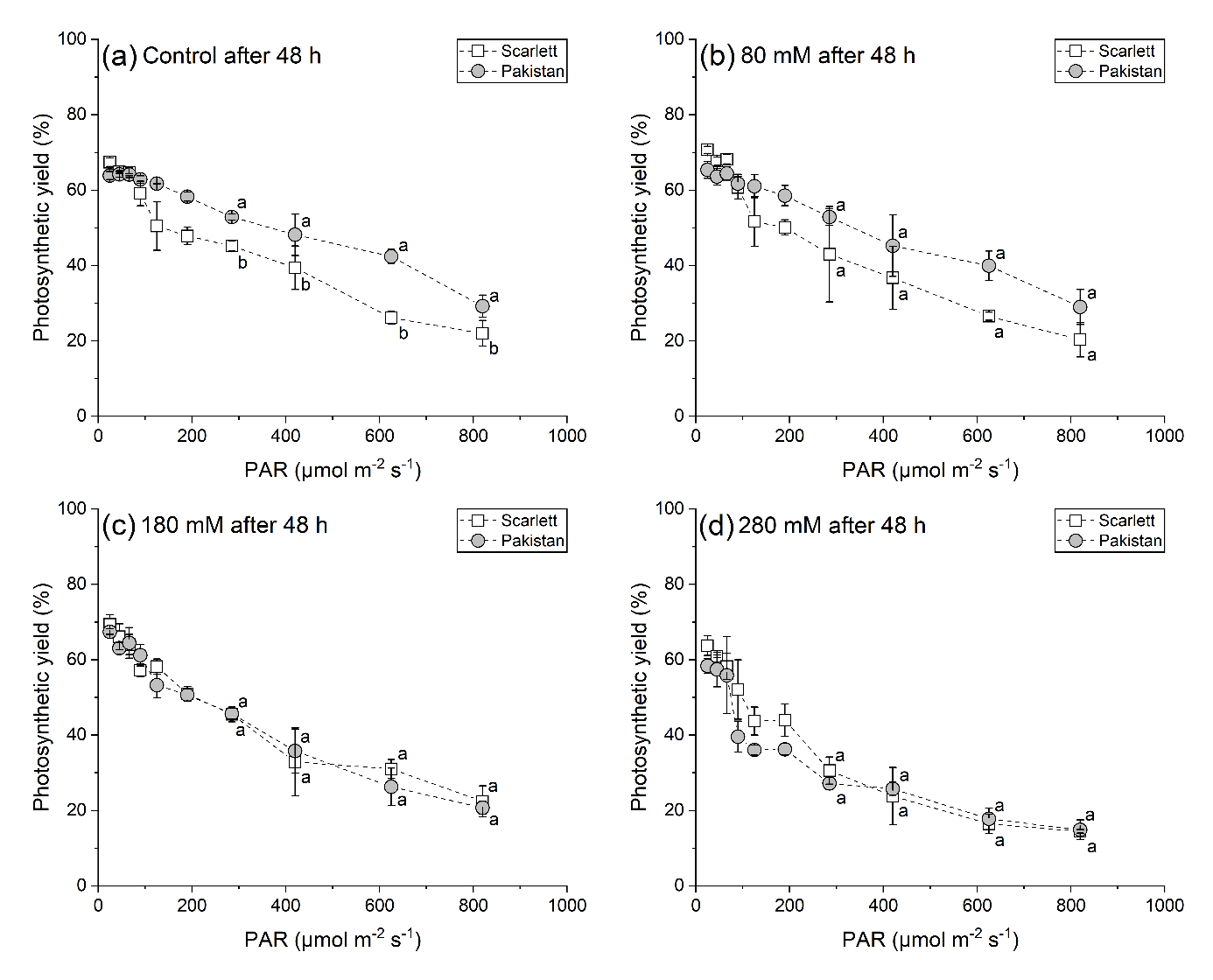


# Fig. S2a-d Short-term physiological reactions of shoots of hydroponically cultivated barley plants subjected to different NaCl stress intensities. The data is identical to Fig. 1c and 1d but plotted differently to visualize potential differences between the accessions. Photosynthetic performance of the first developed leaves was measured non-invasively right after the first 48 hours of control treatment (a), as well as 80 (b), 180 (c), and 280 mM NaCl (d) exposure. Means with standard deviations are shown. Differential letters indicate significant differences at *P* < 0.05; *n* = 3 replicates

#
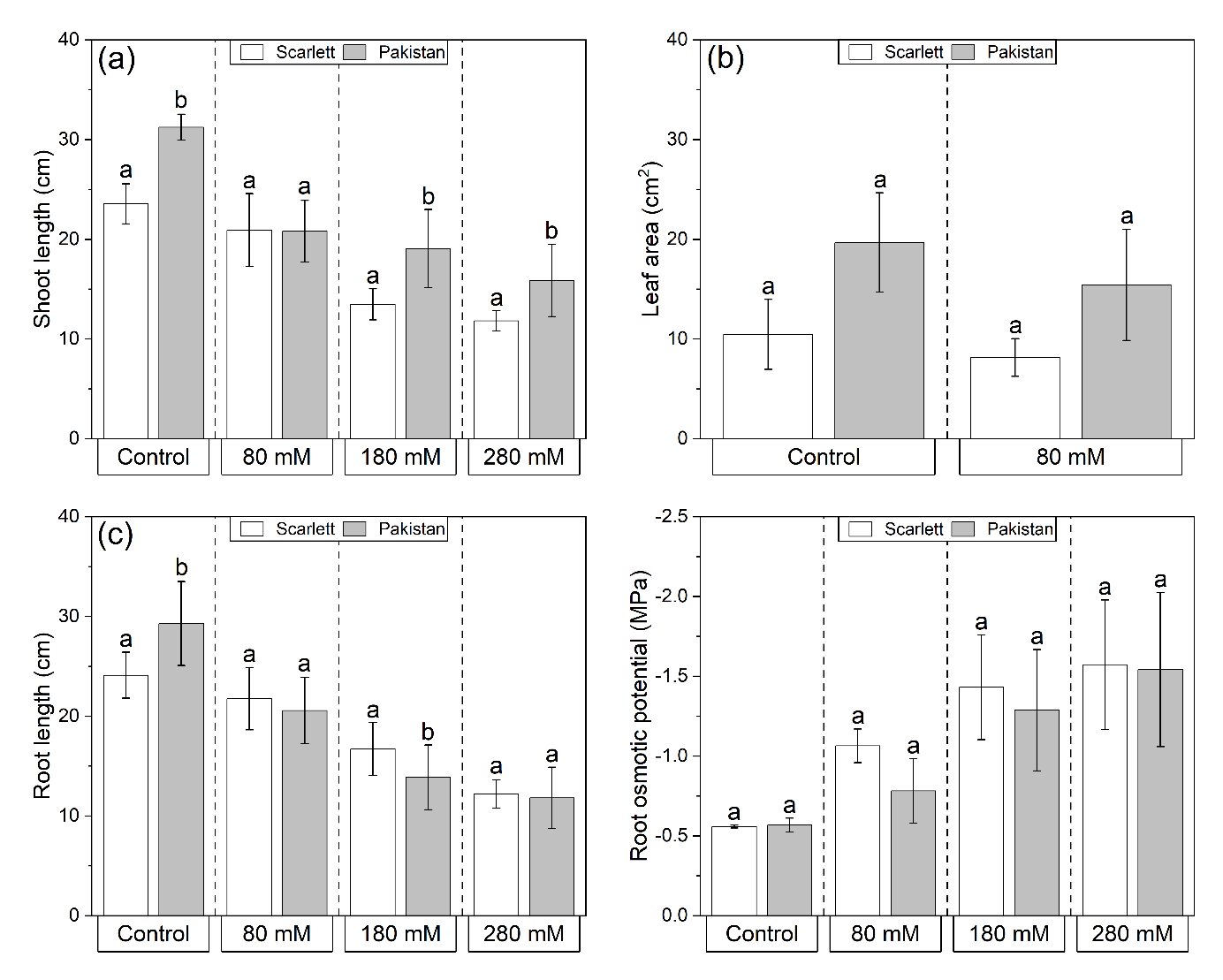


# Fig. S3a-d NaCl stress intensities. The data is identical to Fig. 2 but plotted differently to visualize potential differences between the accessions. Shoot lengths (a), first leaf areas (b), c seminal root lengths (c), and seminal root osmotic potentials (d) were estimated on the day of harvest after 12 days of cultivation, including 6 days of NaCl exposure. Means with standard deviations are shown. Differential letters indicate significant differences at *P* < 0.05; *n* = 7-21 (a), 3 (b), 26-52 (c), and 3 replicates (d)


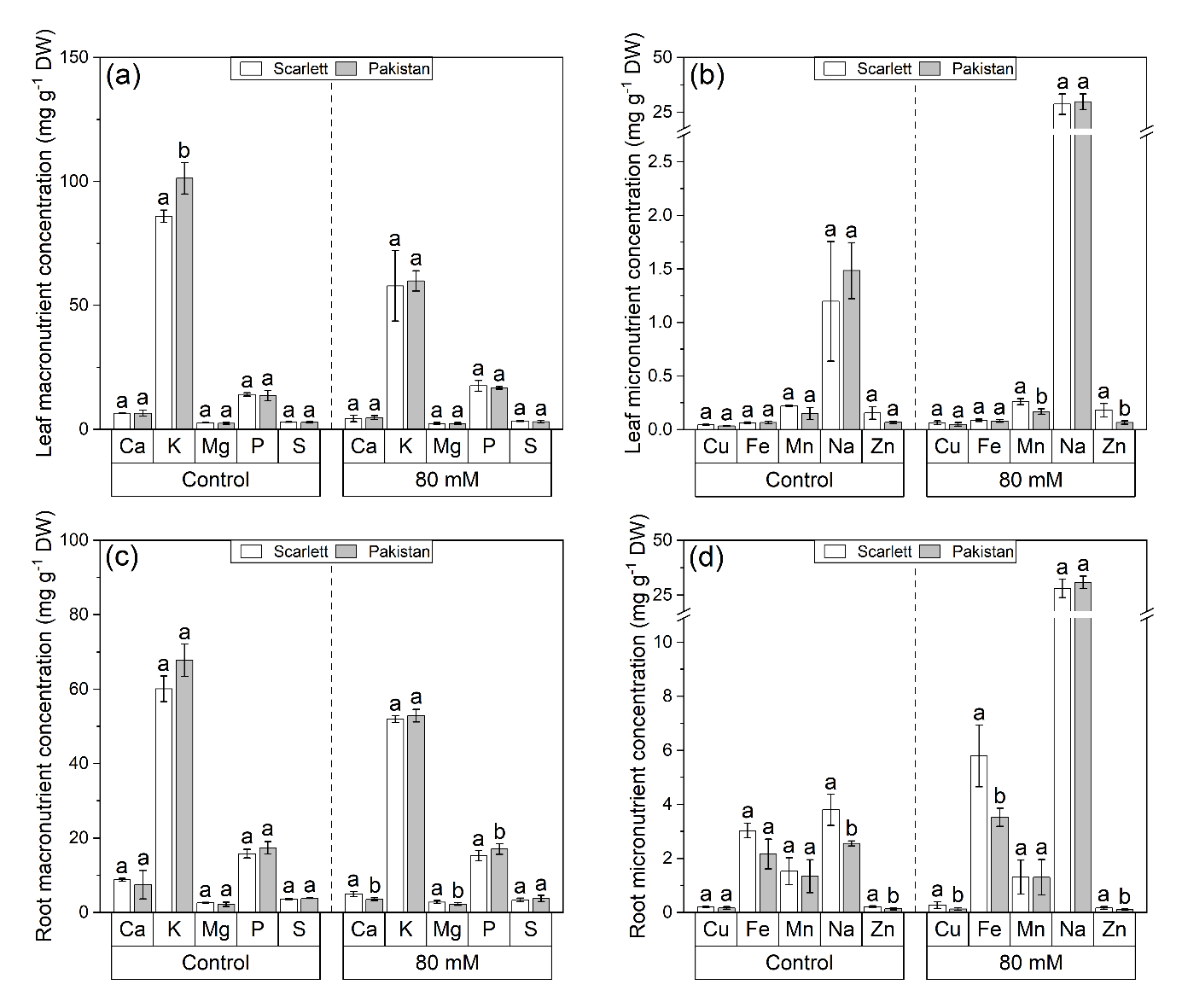


# Fig. S4a-d Ionomes of leaves and roots of hydroponically cultivated barley plants subjected to the lowest tested NaCl stress intensity. The data is identical to Fig. 3 but plotted differently to visualize potential differences between the accessions. Macro (a,c)- and micronutrient (b, d)concentrations of the first leaves and seminal roots were measured after 12 days of cultivation, including 6 days of NaCl exposure. Means with standard deviations are shown. Differential letters indicate significant differences at *P* < 0.05; *n* = 3-6 replicates


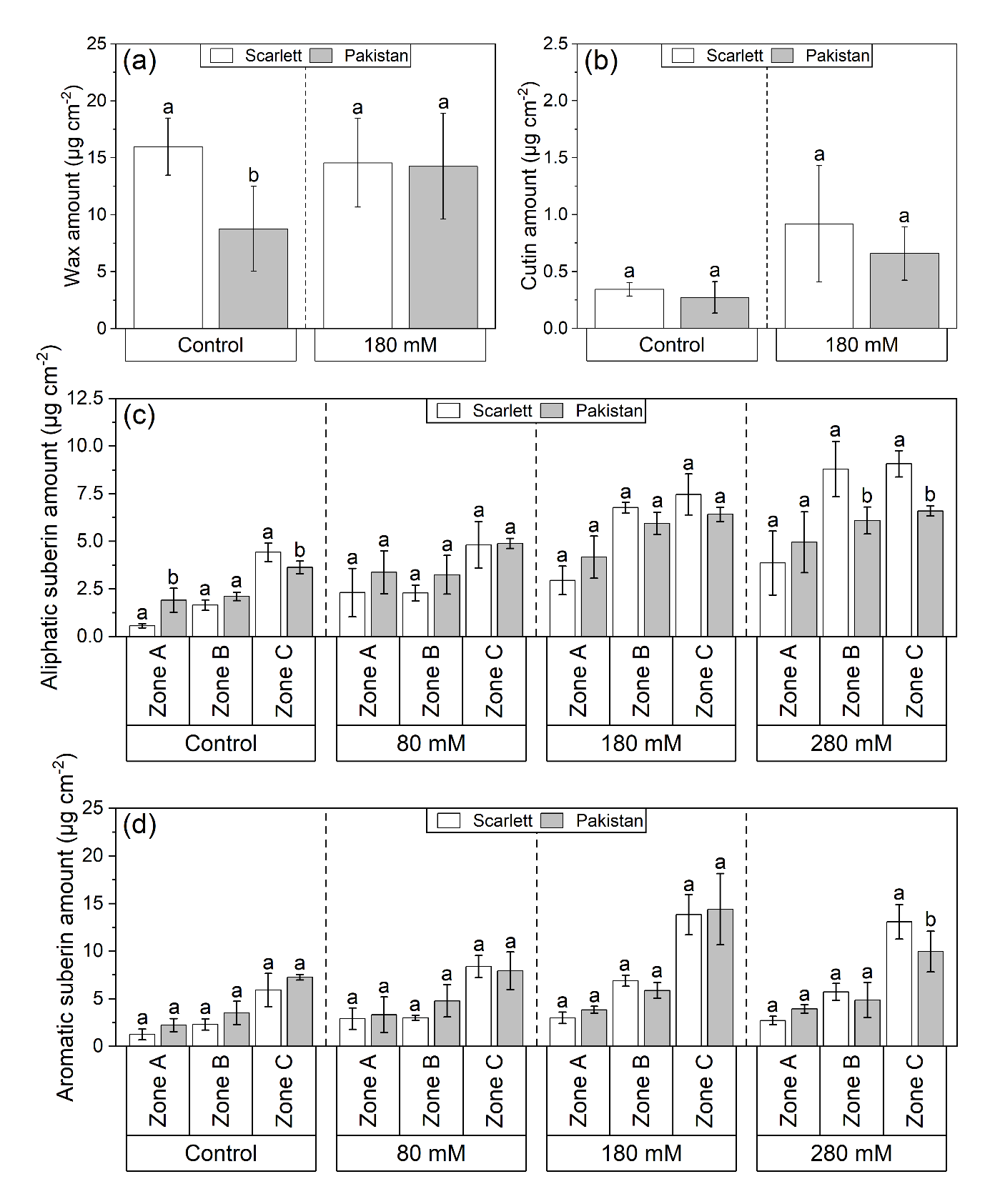


# Fig. S5a-d Chemical analysis of shoot and root apoplastic barriers of hydroponically cultivated barley plants subjected to different NaCl stress intensities. The data is identical to Fig. 5, but plotted differently to visualize potential differences between the accessions. First leaf cuticular wax (a) and cutin amounts (b) were measured for the medium tested NaCl intensity, while seminal root endodermal aliphatic (c) and aromatic suberin (d) amounts were investigated for all NaCl stress intensities. The analysis was performed after 12 days of cultivation, including 6 days of NaCl exposure. The seminal roots were divided into three functional root zones. Only overall amounts are shown here, and more details about monomeric composition are given in the corresponding Figs. S6-S11. Means with standard deviations are shown. Differential letters indicate significant differences at *P* < 0.05; *n* = 3 replicates


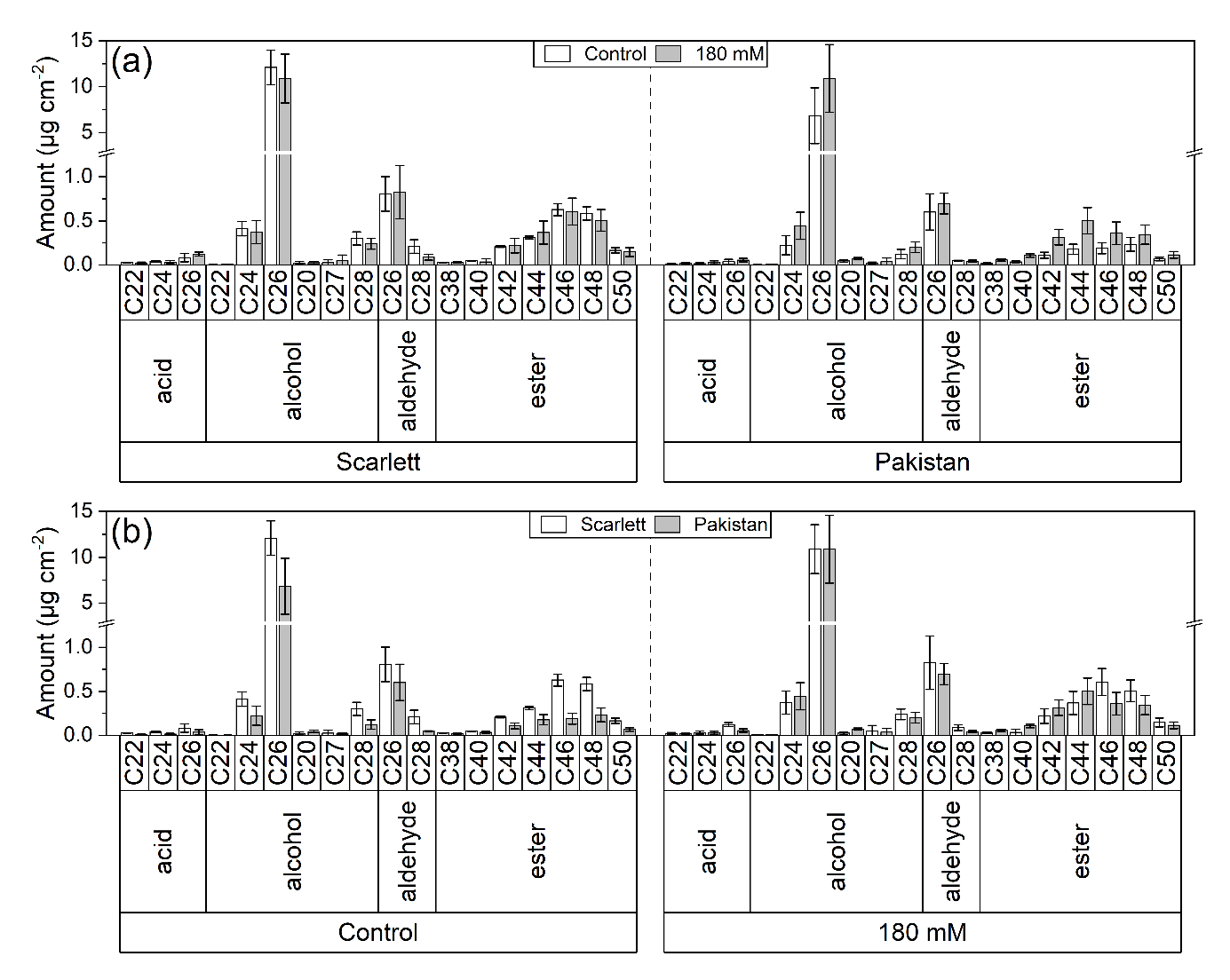


# Fig. S6a,b Chemical analysis of shoot and root apoplastic barriers of hydroponically cultivated barley plants subjected to different NaCl stress intensities. The data is supplementary to Fig. 5 and aims to deliver deeper insights into the monomer composition of the respective apoplastic barrier. Shown is the monomeric wax composition associated with Fig. 5a, sorted for the investigated accessions (a) and the investigated treatments (b). The analysis was performed after 12 days of cultivation, including 6 days of NaCl exposure. Means with standard deviations are shown; *n* = 3 replicates


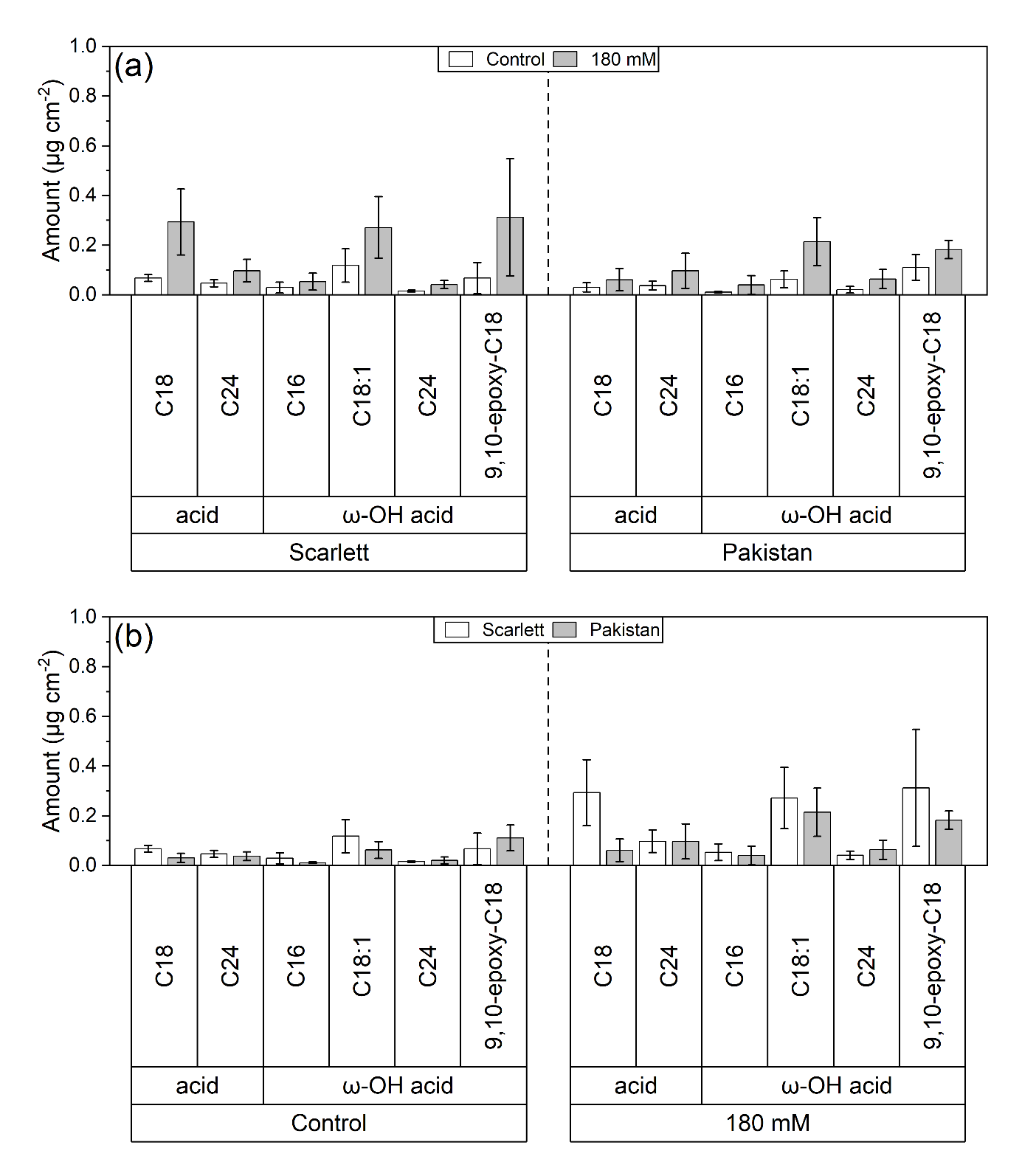


# Fig. S7a,b Chemical analysis of shoot and root apoplastic barriers of hydroponically cultivated barley plants subjected to different NaCl stress intensities. The data is supplementary to Fig. 5 and aims to deliver deeper insights into the monomer composition of the respective apoplastic barrier. Shown is the monomeric cutin composition associated with Fig. 5b, sorted for the investigated accessions (a) and the investigated treatments (b). The analysis was performed after 12 days of cultivation, including 6 days of NaCl exposure. Means with standard deviations are shown; *n* = 3 replicates


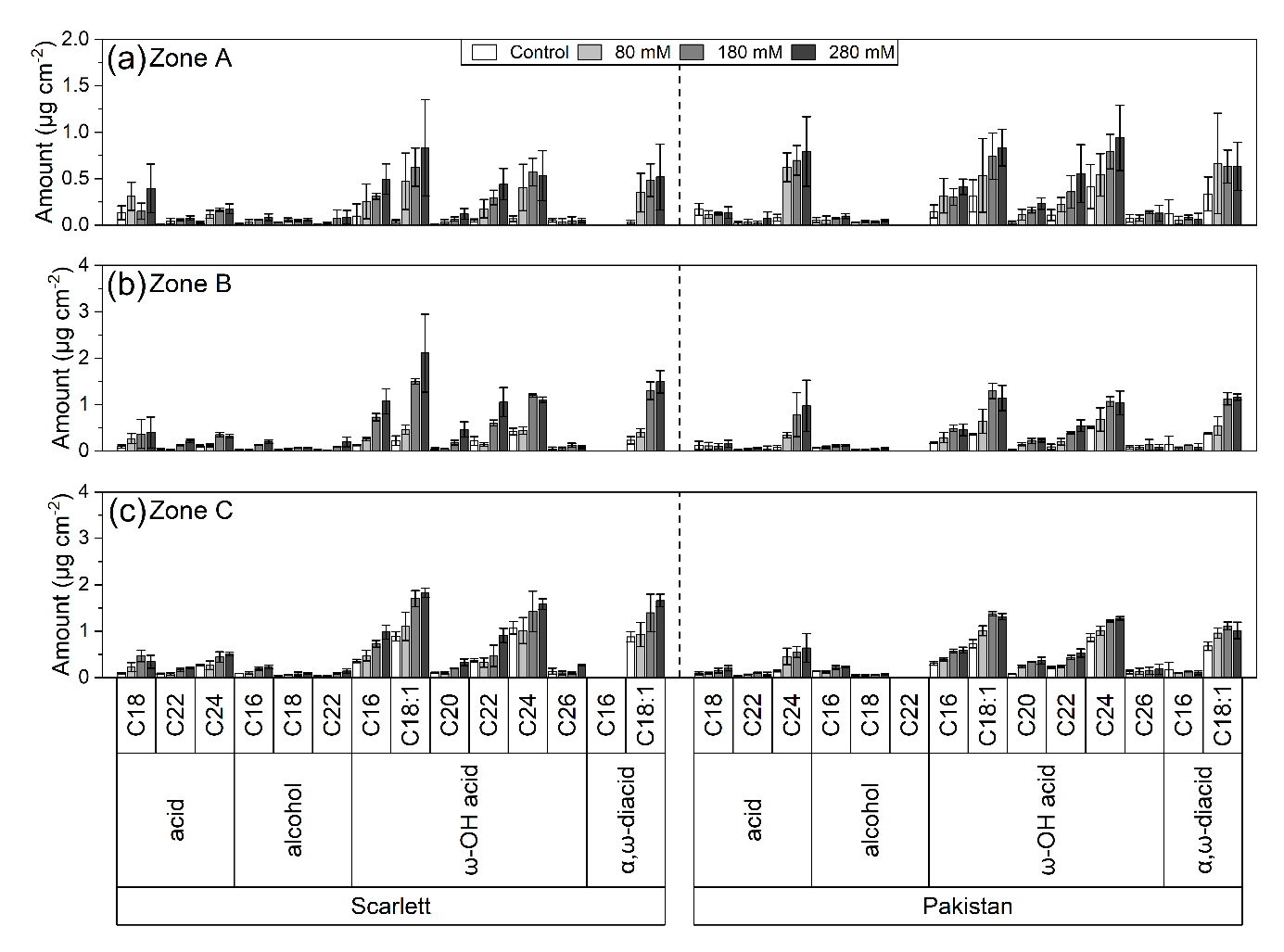


# Fig. S8a-c Chemical analysis of shoot and root apoplastic barriers of hydroponically cultivated barley plants subjected to different NaCl stress intensities. The data is supplementary to Fig. 5 and aims to deliver deeper insights into the monomer composition of the respective apoplastic barrier. Shown is the monomeric aliphatic suberin composition associated with Fig. 5c, sorted for the investigated accessions. The analysis was performed after 12 days of cultivation, including 6 days of NaCl exposure. The seminal roots were divided into three functional root zones: a Zone A, b Zone B, and c Zone C. Means with standard deviations are shown; *n* = 3 replicates


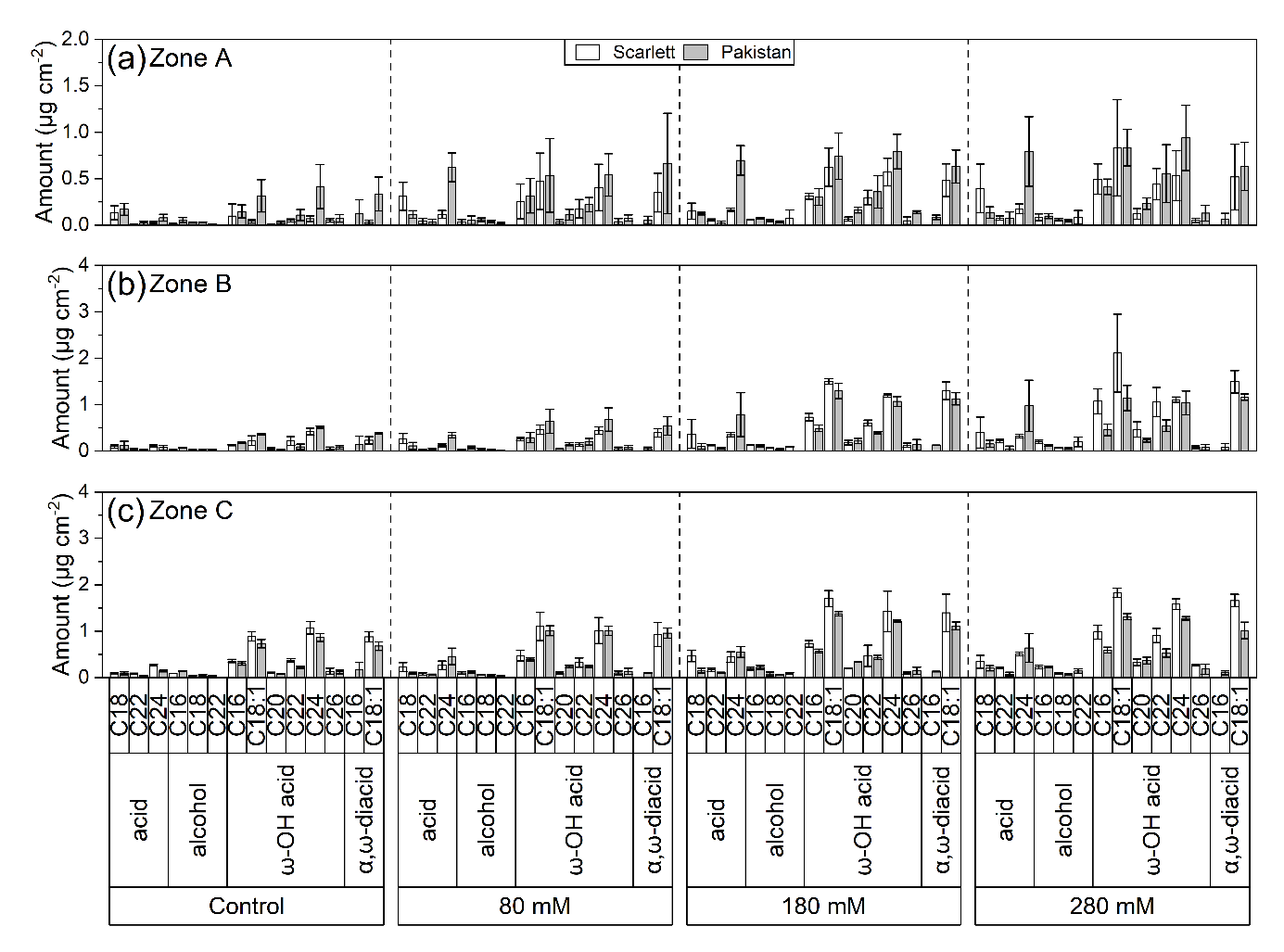


# Fig. S9a-c Chemical analysis of shoot and root apoplastic barriers of hydroponically cultivated barley plants subjected to different NaCl stress intensities. The data is supplementary to Figure 5 and aims to deliver deeper insights into the monomer composition of the respective apoplastic barrier. Shown is the monomeric aliphatic suberin composition associated with Fig. 5c, sorted for the investigated treatments. The analysis was performed after 12 days of cultivation, including 6 days of NaCl exposure. The seminal roots were divided into three functional root zones: a Zone A, b Zone B, and c Zone C. Means with standard deviations are shown; *n* = 3 replicates


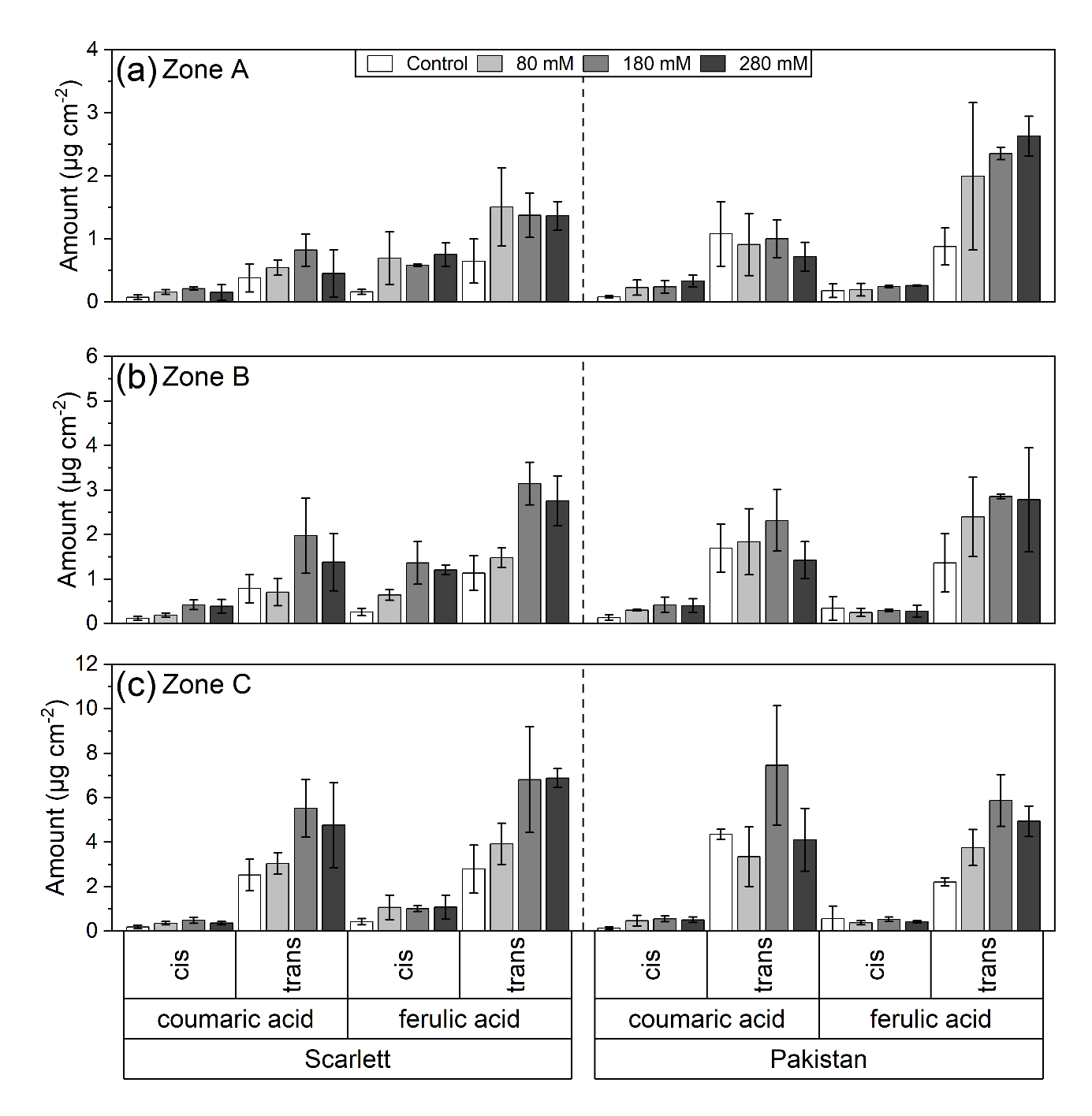


# Fig. S10a-c Chemical analysis of shoot and root apoplastic barriers of hydroponically cultivated barley plants subjected to different NaCl stress intensities. The data is supplementary to Fig. 5 and aims to deliver deeper insights into the monomer composition of the respective apoplastic barrier. Shown is the monomeric aromatic suberin composition associated with Fig. 5d, sorted for the investigated accessions. The analysis was performed after 12 days of cultivation, including 6 days of NaCl exposure. The seminal roots were divided into three functional root zones: a Zone A, b Zone B, and c Zone C. Means with standard deviations are shown; *n* = 3 replicates


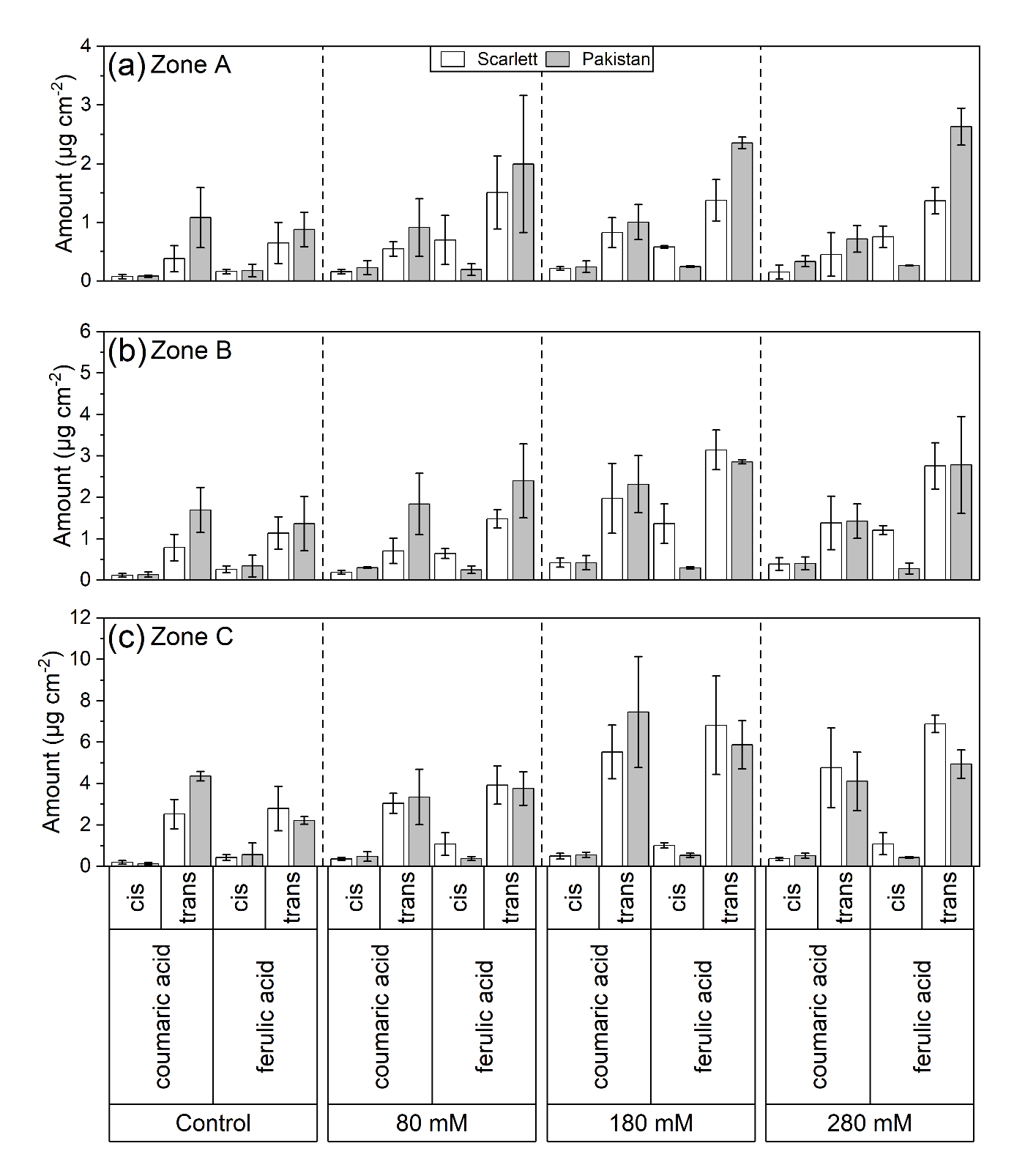


# Fig. S11a-c Chemical analysis of shoot and root apoplastic barriers of hydroponically cultivated barley plants subjected to different NaCl stress intensities. The data is supplementary to Fig. 5 and aims to deliver deeper insights into the monomer composition of the respective apoplastic barrier. Shown is the monomeric aromatic suberin composition associated with Fig. 5d, sorted for the investigated treatments. The analysis was performed after 12 days of cultivation, including 6 days of NaCl exposure. The seminal roots were divided into three functional root zones: a Zone A, b Zone B, and c Zone C. Means with standard deviations are shown; *n* = 3 replicates
